# Prospective Pilot Study of Lavender Terpenoid Topical Formulation in Psoriasis Patients with Chronic Kidney Disease: Clinical and Inflammatory Outcomes

**DOI:** 10.3390/biomedicines14030552

**Published:** 2026-02-27

**Authors:** Corina Moisa, Ioana Adela Rațiu, Adrian Topală, Vladilena Gîrbu, Aculina Arîcu, Veaceslav Kulcițki, Mirela Cacuci, Cristian Adrian Rațiu, Laura Maghiar, Teodor Andrei Maghiar, Mariana Ganea

**Affiliations:** 1 Faculty of Medicine and Pharmacy, University of Oradea, 1st December Square 10, 410073 Oradea, Romania; cmoisa@uoradea.ro (C.M.); cristian.ratiu@didactic.uoradea.ro (C.A.R.); laura.maghiar@uoradea.ro (L.M.); teodormaghiar@yahoo.com (T.A.M.); mganea@uoradea.ro (M.G.); 2 Nephrology Department, Emergency Clinical Hospital Bihor County, 410087 Oradea, Romania; 3 Institute of Chemistry, Moldova State University, 3 Academiei Street, MD-2028 Chișinău, Moldova; cbf20topala.adrian@gmail.com (A.T.); vladilena.girbu@sti.usm.md (V.G.); aculina.aricu@sti.usm.md (A.A.); 4 Pelican Hospital, 2 Corneliu Coposu Street, 410450 Oradea, Romania; drcacucimi@yahoo.com; 5 Psychoneuroscience and Recovery Department, University of Oradea, 1st December Square 10, 410028 Oradea, Romania; 6 Clinical County Emergency Hospital Oradea, 65 Gh. Doja Street, 410169 Oradea, Romania; 7 Surgery Department, University of Oradea, 1st December Street, 410028 Oradea, Romania

**Keywords:** psoriasis, triterpenoid acids, *Lavandula angustifolia*, chronic kidney disease, urinary albumin/creatinine ratio (uACR), inflammation, neutrophil–lymphocyte ratio (NLR), hs-CRP/albumin

## Abstract

**Background/Objectives**: Psoriasis is a chronic, recurrent inflammatory skin disorder with a steadily rising prevalence worldwide. Current therapeutic strategies include topical and systemic treatments selected based on disease severity and associated comorbidities; however, no therapy provided a definitive cure. Topical therapy is associated with a lower risk of systemic adverse effects, although the altered skin barrier observed in psoriasis may significantly reduce the bioavailability of active pharmaceutical ingredients. The aim of this study was to develop and evaluate the efficacy of a topical formulation containing triterpenoid acids derived from lavender extract (*Lavandula angustifolia* Mill.) as a potential adjunctive approach for symptom management in patients with psoriasis and chronic kidney disease (CKD). **Methods**: Following removal of the volatile oil, oleanolic, ursolic, pomolic, and rosmarinic acids were identified and quantified. The preparation was analyzed in terms of organoleptic properties, colloidal stability, pH determination, and rheological characteristics. The clinical study included 18 patients (both sexes) aged 28 to 71 years, with psoriasis and CKD of varying etiologies. Urinary albumin/creatinine ratio (uACR) and estimated glomerular filtration rate (eGFR) were used as renal biomarkers, while high-sensitive C-reactive protein (hs-CRP), hs-CRP/albumin, and neutrophil–lymphocyte ratio (NLR) were selected as inflammatory biomarkers. Laboratory assessments were performed at baseline and after 60 days of topical treatment with the lavender extract-based formulation. Clinical outcomes were evaluated using validated measures of psoriasis severity and patient impact, including the Psoriasis Area and Severity Index (PASI), the Investigator Global Assessment (IGA), and the Dermatology Life Quality Index (DLQI). **Results**: The formulation contained 1.4% rosmarinic acid and up to 8% ursolic acid (extract mass) and demonstrated good stability, a pH of 5.5, favorable local tolerability, antiproliferative activity, reduction in pruritus, and no treatment-related adverse effects. Efficacy and tolerability scores showed statistically significant improvements at 60 days (T2) after topical terpenoid administration compared with baseline (T0): PASI (0.722 ± 0.714 vs. 2.044 ± 0.690 at T0, *p* < 0.001), DLQI (3.889 ± 1.997 vs. 13.333 ± 3.361 at T0, *p* < 0.001), and IGA (0.556 ± 0.616 at T2 vs. 2.167 ± 0.618, *p* < 0.001). uACR decreased significantly over the study period (308.714 ± 240.782 after 60 days vs. 379.78 ± 308.81 at T0, *p* < 0.001), while eGFR values remained similar at baseline and follow-up. All evaluated inflammatory biomarkers showed significant reductions: hs-CRP (4.33 ± 2.127 at T2 vs. 9.7 ± 10.045 at T0, *p* < 0.009), hs-CRP/albumin ratio (0.105 ± 0.052 at T2 vs. 0.241 ± 0.225, *p* < 0.011), and NLR (2.154 ± 2.171 vs. 2.253 ± 0.256 at baseline, *p* = 0.027). Linear regression analysis identified no predictors of responsiveness to topical triterpenoid therapy in patients with psoriasis and CKD. **Conclusions**: This topical lavender extract-based formulation showed promising clinical and anti-inflammatory effects and favorable local tolerability in this pilot cohort of psoriasis patients with CKD. However, these findings should be considered preliminary and require confirmation in larger randomized controlled studies.

## 1. Introduction

Psoriasis is a chronic inflammatory dermatosis with multiple clinical manifestations, the most common being plaque psoriasis, characterized by erythematous plaques that typically affect the elbows, knees, scalp, and lower back [1]. The hallmark clinical features of psoriasis include erythema and scaling, resulting from extensive infiltration of inflammatory T cells into both the dermis and epidermis [2].

Following exposure to internal or external stimuli, dendritic cells in the dermis and Langerhans cells in the epidermis become activated and migrate to regional lymph nodes, where they promote T-cell activation through CD45RA+ T lymphocytes. This process leads to the release of pro-inflammatory cytokines, including tumor necrosis factor alpha (TNF-α) and interleukin-6 (IL-6), which directly drive keratinocyte hyperproliferation. Collectively, these mechanisms contribute to the development and persistence of psoriatic skin lesions [3]. Systemic inflammation associated with psoriasis affects multiple organs, with the kidneys being particularly susceptible to immune-mediated damage involving nuclear factor kappa B (NF-κB) signaling pathways [4]. Increased angiotensinogen production induced by TNF-α may lead to elevated blood pressure, vascular injury, and subsequent impairment of renal filtration capacity, reflected by a reduced estimated glomerular filtration rate (eGFR) [5,6]. Pro-inflammatory cytokines, particularly interleukin-17 (IL-17), have been associated with psoriasis severity, immune-mediated glomerulonephritis, renal dysfunction, and decreased eGFR values [7,8].

Several epidemiological studies have reported that up to 8% of patients with psoriasis may develop chronic kidney disease. However, no statistically significant differences in psoriasis incidence have been observed between patients with and without chronic kidney disease, even in large-scale cohorts of more than one million individuals [9]. In contrast, extensive studies by Wan et al. and Lee et al. had demonstrated that moderate to severe psoriasis is associated with an increased risk of chronic kidney disease, particularly in advanced stages [10,11].

Given the chronic nature of psoriasis, clinical management primarily aims to alleviate symptoms, improve patients’ quality of life, and prevent disease recurrence. Therapeutic regimens include oral agents such as immunosuppressants, retinoids, and corticosteroids; injectable drugs (e.g., methotrexate, azathioprine, and hydroxyurea); and topical therapies, including corticosteroids and calcineurin inhibitors, particularly in patients with mild to moderate disease severity [12,13,14,15].

Although these treatments improve symptoms, they do not provide a definitive cure. Moreover, long-term use is frequently associated with adverse effects, including immunosuppression, skin atrophy, organ toxicity, and increased susceptibility to infections. Consequently, plant-derived products with antioxidant and anti-inflammatory properties and a favorable safety profile have emerged as promising alternatives to conventional therapies. Indeed, phytotherapeutic approaches have demonstrated therapeutic potential in various conditions and have gained increasing attention over recent decades [16,17].

Accumulating evidence supports the hypothesis that bioactive compounds derived from medicinal plants may counteract key pathophysiological mechanisms involved in psoriasis while alleviating clinical symptoms. Natural compounds with documented efficacy in psoriasis include alkaloids, anthraquinones and their derivatives, coumarins, flavonoids, phytosterols, polyphenolic acids, polysaccharides, and terpenoids [18,19,20,21,22,23,24].

Clinical studies conducted over the past decade have predominantly involved patients with mild-to-moderate psoriasis treated with topical formulations containing natural compounds. Puaratanaarunkon et al. evaluated 51 patients with mild psoriasis treated with a 2.5% cannabidiol ointment applied twice daily for 12 weeks, reporting a significant reduction in clinical symptoms [25]. Kolahdooz et al. conducted a clinical study of five patients with mild-to-moderate psoriasis treated with a 0.1% curcumin gel applied twice daily for four weeks, resulting in reduced scaling and pruritus [26]. Bocheńska et al. administered genistein in film-coated tablets to 30 patients with mild-to-moderate psoriasis and observed improvements in both clinical and biochemical scores [27]. Indirubin-containing ointments were applied to psoriatic lesions in 25 patients, resulting in symptom improvement after an eight-week treatment period [28,29].

Lavender contains numerous chemical compounds, including organic acids belonging to the triterpenoid group: oleanolic acid, ursolic acid, pomolic acid, and rosmarinic acid.

Oleanolic acid has multisystem effects. At the metabolic level, it helps reduce atherosclerosis by regulating dyslipidemia and prevents diabetes mellitus by protecting pancreatic cells. In addition, it has demonstrated antibacterial, antifungal, anti-inflammatory, and anticancer effects [30]. Pomolic acid exerts an endothelium-dependent vasorelaxant effect and has been studied for its potential for treating arterial hypertension [31]. Ursolic acid predominantly exerts anticancer effects at the multiorgan level by influencing cellular metabolism and through anti-inflammatory, antioxidant, and antiviral effects [32]. Rosmarinic acid has antioxidant, preventive effects against Alzheimer’s disease, antitumor effects studied in colon neoplasms, protective effects against skin aging, and antithrombotic effects [33,34,35,36].

Considering the therapeutic potential, high availability, and minimal adverse effects, there is growing interest in incorporating plant-derived compounds into effective topical formulations as viable alternatives for psoriasis management [37]. Although clinical studies have reported the inclusion of triterpenoids in topical formulations for the treatment of mild psoriasis [38], to the best of our knowledge, no clinical studies have investigated triterpenoids isolated from lavender or demonstrated their efficacy in psoriasis treatment.

Given the chronic course of psoriasis, the variability of clinical presentation, and the disproportionate impact that even mild-to-moderate disease can have on patients’ quality of life, objective assessment of disease severity and therapeutic response is essential. PASI is a validated composite index that quantifies psoriasis severity by integrating lesion extent and key morphological features (erythema, induration, and scaling). IGA provides a clinician-rated global severity grade on an ordinal scale, enabling rapid assessment of overall plaque activity. DLQI is a standardized patient-reported outcome measure that captures the functional and psychosocial burden of skin disease and complements clinician-based severity metrics [39,40,41,42]. In this context, the use of validated clinical scoring systems, such as the Psoriasis Area and Severity Index (PASI), Investigator’s Global Assessment (IGA), and Dermatology Life Quality Index (DLQI), allows standardized evaluation of disease activity while incorporating patient-reported functional and psychosocial outcomes.

The aim of this study was to formulate a topical preparation containing triterpenoids isolated from an extract of *Lavandula angustifolia* and to evaluate its efficacy in improving clinical symptoms in patients with mild-to-moderate psoriasis. This formulation was developed as a safe alternative to conventional topical therapies, with therapeutic outcomes assessed using validated clinical scoring systems to quantify disease severity and quality-of-life impact.

## 2. Materials and Methods

### 2.1. Pharmacological Study

**Figure 1:**
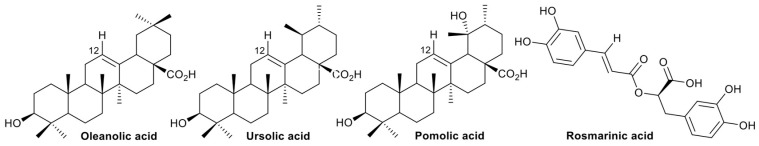
Chemical structure of organic acids quantified in the lavender extract by quantitative 2D-NMR spectroscopy.

Preparation of *Lavandula angustifolia* Extract (Figure 1).

### 2.2. General

Ultrasound-assisted extraction was performed in a Raypa UC-150 ultrasonic bath (Terrassa-Barcelona, Spain) at a fixed power of 400 W and an operating frequency of 35 kHz in complete-wave mode. For evaporation of extracts, a Hei-VAP Value G3 (Heidolph Instruments GmbH & Co. KG, Schwabach, Germany) was used with the bath temperature set to 40 °C under vacuum. NMR Spectra have been recorded on a Bruker-Avance-III spectrometer (400.13 and 100.61 MHz) in dimethyl sulfoxide (DMSO)-d_6_ (ca. 30 mg of material in 0.75 mL of DMSO-d_6_); δ in ppm relative to DMSO-d_6_ as internal standard (δ(H) 2.50 and δ(C) 39.51), J in Hz. Gravimetric operations during calibration curve plotting were performed on an MX5 Mettler Toledo precision microbalance (Max 5.1 g, d = 1 μg).

### 2.3. Reagents and Chemicals

All reagents, including reagent-grade oleanolic acid (OA) and nuclear magnetic resonance (NMR) solvents, have been purchased from Sigma-Aldrich (St. Louis, MO, USA) and used as received. Ursolic (UA), pomolic (PA), and rosmarinic (RA) acids have been isolated from the lavender extract as previously described [24,43]. The identification of UA, PA, and RA was based on ^1^H and ^13^C NMR spectra; their contents in the reference samples were determined by ^1^H NMR using methyl 4-nitrobenzoate as an internal standard (IS).

### 2.4. Plant Material

Lavender plant material (*Lavandula angustifolia* Mill.) was collected during the harvest period (July 2023) onsite of the industrial production facility of Cobusca Nouă essential oil company, Republic of Moldova (46°55′52.68″, 29°12′24.84″), in the wet form immediately after steam distillation and essential oil separation. It was dried in a shed with continuous ventilation at ambient temperature until a constant weight. The dried material was stored in Kraft paper bags in well-ventilated areas at ambient temperature. The separation of flowers from stalks was performed manually. The flowers have been used in their intact form for extraction.

### 2.5. Extraction

The plant material (100 g) was placed in an Erlenmeyer flask, and food-grade (95%) ethanol (800 mL) was added. The flask was immersed in an ultrasonic bath for irradiation at 60 °C. Extraction included sequential irradiation (15 min) and passive maceration (30 min), repeated 3 times at the same temperature. The hot extracts were decanted from the vegetal material and filtered through a Whatman No. 1 paper filter. The solvent was distilled off on a rotary evaporator. The crude extracts were dried under vacuum (2 Torr) for 60 min at room temperature and kept at −20 °C under a nitrogen atmosphere. The yield of the extracts was calculated in grams of extract per 100 g of vegetal material dry weight. The content of individual triterpenoid acids in the obtained extracts was calculated as a percentage of the extract’s dry weight.

### 2.6. qNMR Analysis of Organic Acids in Lavender Extract

Quantitative determination of organic acids in the lavender extract was performed using two-dimensional nuclear magnetic resonance (2D NMR) analysis based on the heteronuclear single quantum coherence (HSQC) experiment, as was described previously for triterpenoid acids [43]. Data acquisition parameters for ^1^H NMR spectra were: zg30 excitation pulse, spectral width 8224 Hz, 64K real data points, acquisition time 3.98 s, relaxation delay 25 s. ^1^H–^13^C HSQC experiments were acquired with a spectral width of 4401 Hz in the F_2_ dimension and 18,112 Hz in the F_1_ dimension, acquisition time was 0.11 s, relaxation delay was 2.5 s, the t_1_ dimension was zero-filled to 1K real data points, and 4 scans were acquired per increment. T_1_ was measured using the null-point method based on the inversion recovery pulse sequence for all individual organic acids and IS. NMR data processing, including T_1_, signal-to-noise (s/n), and manual 2D cross-peak integration, was performed using TopSpin 3.6.4 NMR software (Bruker, Rheinstetten, Germany).

**Figure 2:**
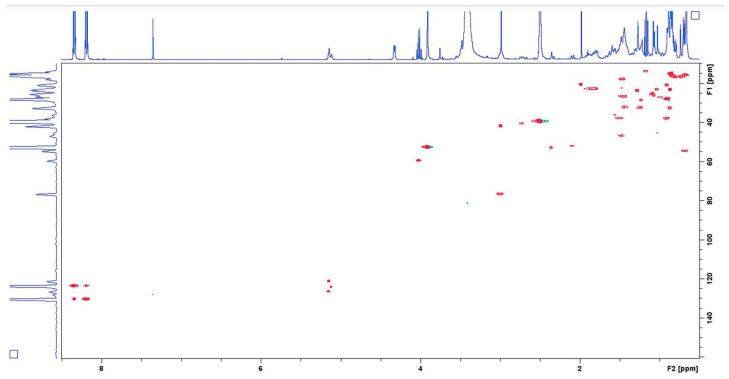
HSQC NMR spectrum of an artificial mixture of oleanolic, ursolic and pomolic acids containing internal standard methyl 4-nitrobenzoate.

**Figure 3:**
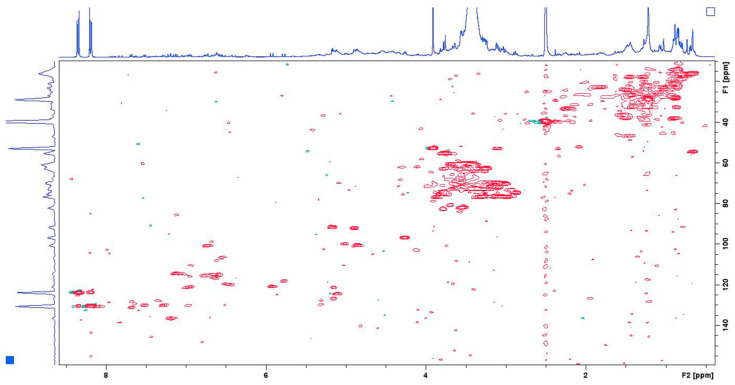
HSQC NMR spectrum of lavender extract containing methyl 4-nitrobenzoate as internal standard.

Quantitative analysis was performed using five-point calibration curves for each organic acid. To plot the individual calibration curves, 30 mM solutions of each organic acid in DMSO-d_6_ were prepared, and sequential portions of the internal standard, dissolved in the same solvent, were added. The calibration curves were obtained by plotting the ratio between integrals of C (12)-H (for OA-5.15 ppm, 121.44 ppm, UA-5.12 ppm, 124.52 ppm, PA-5.16 ppm, 126.74 ppm) or C (7)-H (for RA-7.44 ppm, 145.73 ppm) and those of the C (3)-H for IS (8.35 ppm, 123.89 ppm) versus molar ratio between OA, UA, PA or RA and the IS. The regression coefficient values were 0.9953 for OA and UA, 0.9995 for PA, and 1.0 for RA. To measure the limits of detection and quantification, the s/n ratio was calculated from HSQC experiments. Standard solutions of pure organic acids were prepared in concentrations ranging from 1.1 mM to 11 mM, and 2D NMR HSQC spectra were recorded in the presence of IS. Each quantitative HSQC spectrum required 45 min to collect (4 scans). LOD (s/n > 4) was determined at 2.2 mM and LOQ (s/n > 10) at 4.4 mM of each organic acid. All measurements were performed in triplicate (on three different extracts of the same sample) (Figure 2 and Figure 3).

#### Preparation of the Bioadhesive Topical Formulation

The composition of the topical formulation is presented in Table 1. Paraffin oil, eucerin, and petrolatum were purchased from SC Vitamar Import Export (Bucharest, Romania) and were accompanied by certificates of conformity.

**Figure 4:**
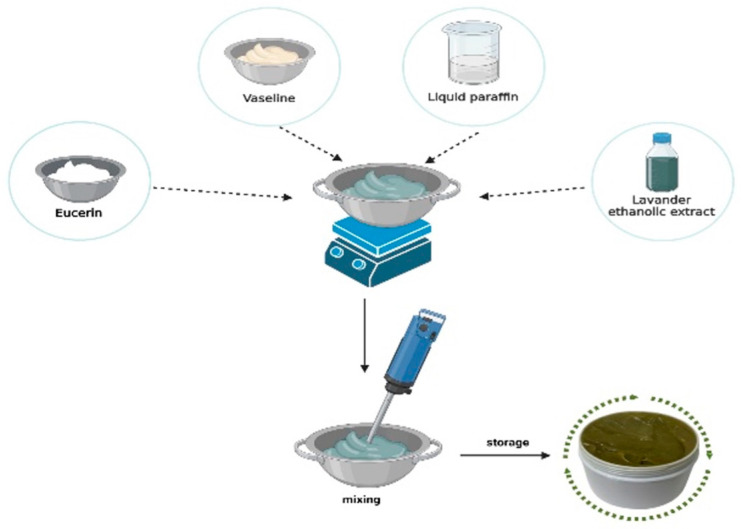
Preparation of the bioadhesive topical formulation containing lavender extract.

The preparation of the lavender extract-based ointment was carried out in two stages. In the first stage, the ointment base was prepared by gradually melting and homogenizing the lipid-phase components. The second stage involved incorporating the pharmacologically active ingredient into the obtained ointment base. Following homogenization, a semisolid preparation with an olive-green appearance was obtained (Figure 4)

### 2.7. Characterization of the Bioadhesive Topical Formulation

#### 2.7.1. Organoleptic Evaluation

Organoleptic evaluation is an important part of the preliminary assessment of topical formulations, as it provides relevant information on the physical appearance, homogeneity, color, and odor of the resulting preparations. Given their topical application, these characteristics have a substantial impact on product acceptability, patient compliance, and overall perception of formulation quality.

#### 2.7.2. pH Determination

The pH of each formulation was determined using a Hanna Instruments Inc. 8417 pH meter (Woonsocket, RI, USA). The instrument was calibrated prior to analysis, and the electrode was immersed directly into the samples for measurement.

#### 2.7.3. Colloidal Stability of Pharmaceutical Preparations

The colloidal stability of the pharmaceutical preparations was evaluated by a centrifugation method using a Hettich Universal 320 R centrifuge (Tuttlingen, Germany). A volume of 30 mL of each sample was placed in conical polyethylene tubes and centrifuged at 6000 rpm for 20 min at laboratory temperature (25 ± 1 °C).

Time-dependent phase separation during centrifugation was simulated, and the phase stability of the formulations was assessed using the stability index (SI). The SI was calculated according to the following equation:

$$SI\left(\%\right)=\left(1-\frac{V_{2}}{V_{1}}\right)\times 100$$

where $V_{1}$ represents the total volume of the formulation, and $V_{2}$ denotes the volume of the separated oil phase (supernatant) after centrifugation.

#### 2.7.4. Rheological Analysis

The apparent viscosity of the formulations was determined using a rotational viscometer (Lamy Rheology Instruments, Champagne-au-Mont-d’Or, France) equipped with a coaxial cylindrical measuring system (MS-DIN 33). To evaluate flow behavior, viscosity was measured over a wide range of shear rates (1–100 s^−1^) to capture both low-shear conditions (rest/storage) and high-shear conditions (application/spreading).

At each shear rate, viscosity was measured after signal stabilization, yielding viscosity–shear rate datasets. The experimental data were processed in MATLAB (MathWorks, Natick, MA, USA) to generate graphical representations of viscosity as a function of shear rate.

### 2.8. Clinical Study

Kidney Disease: Improving Global Outcomes (KDIGO) guidelines recommend using two fundamental biomarkers to assess renal impairment: microalbuminuria, a marker of renal injury, and GFR as a marker of renal function. Microalbuminuria is closely associated with glomerular endothelial dysfunction. Microalbuminuria correlates with glomerular endothelial dysfunction. It correlates with systemic inflammatory activity and is associated with increased cardiovascular risk and all-cause mortality in CKD. Reducing systemic inflammation may favorably influence microalbuminuria and preserve glomerular filtration rate. The present study aimed to evaluate the potential impact of topical psoriasis therapy with lavender extract on CKD progression.

Study design: Given the relatively limited systemic treatment options for psoriatic lesions in patients with CKD, we conducted a prospective, interventional pilot study of a before-and-after design involving 18 patients with CKD of various etiologies and stages, monitored at the Bihor County Clinical Hospital Pelican. This proof-of-concept study followed patients from 1 June 2025 to 31 August 2025, with assessments at treatment initiation and after 60 days of topical treatment with a cream containing lavender extract. For consistency throughout the manuscript, the assessment before treatment initiation was designated baseline (T0), and the follow-up assessment after 60 days of topical treatment was designated T2. Although the study posed a potentially low risk, it involved a relatively vulnerable population. The study was conducted in accordance with the ethical principles of the Declaration of Helsinki of the World Medical Association (WMA) for research involving human subjects [44] and received approval from the Hospital Ethics Committee (Approval No. 1225/27 May 2025). All patients included in the study had signed an informed consent. Data were collected from patients’ records.

Topical treatment protocol: Patients were directed to apply the topical lavender extract formulation twice daily for 60 days as a thin layer directly onto the affected psoriatic regions, using a fingertip-unit (FTU) dosing method (i.e., approximately consistent amount applied from the fingertip) sufficient to ensure complete lesion coverage. The exact dose per unit skin surface area (e.g., g/cm^2^) was not quantified in this pilot study.

Study objectives: The study aims to assess the renal safety and clinical efficacy of a topical lavender extract-based formulation by measuring changes in renal function parameters, inflammatory biomarkers, and validated psoriasis severity and quality-of-life scores (PASI, IGA, DLQI) following 2 months of topical treatment.

#### 2.8.1. Primary Objective

To evaluate the clinical efficacy of the lavender-based cream in the treatment of psoriasis in patients with CKD.

Primary Endpoint: Reduction in PASI score from baseline to 60 days following the application of a topical preparation containing triterpenoids isolated from *Lavandula angustifolia* extract.

Secondary Endpoints: Reduction in DLQI and IGA scores at 60 days following topical application of triterpenoids.

#### 2.8.2. Secondary Objectives

(a) To evaluate the renal safety of the topical treatment;

(b) To assess changes in systemic inflammatory biomarkers;

(c) To analyze the relationship between clinical response and biological parameters.

Secondary Endpoints: (a) Stability of eGFR, microalbuminuria, and uric acid as biomarkers of renal involvement; (b) Changes in inflammatory biomarkers, including NLR and CRP/albumin ratio.

Inclusion criteria:

(a) patients aged > 18 years;

(b) patients with documented CKD.

Exclusion criteria:

(a) patients with immunosuppressive/immunomodulatory treatment;

(b) patients with acute decline of renal function;

(c) patients with a need for systemic anti-psoriasis treatment.

Data sources and collection:

Data was collected from medical records before (T0) and after the 2 months (T2) of topical dermatological treatment and included:

(a) demographic findings: age, gender, duration of CKD;

(b) underlying kidney disease;

(c) creatinine and uric acid levels (mg/dL), eGFR (CKD-EPI 2021), microalbuminuria before and after the treatment;

(d) hs-CRP, NLR, and CRP/albumin ratio before and after the topical treatment.

Serum creatinine, uric acid, and the urinary albumin-to-creatinine ratio (uACR) were determined using the Cobas 8000 analyzer (Roche, Basel, Switzerland), module COBAS c 702. Quantification of creatinine, uric acid, and albumin was performed by spectrophotometry; hs-CRP was measured using an immunoturbidimetric assay. Complete blood counts were performed on the SYSMEX XN-1000 hematology analyzer (Sysmex, Hamburg, Germany), using fluorescence flow cytometry with hydrodynamically focused impedance technology.

(e) Clinical assessment of psoriasis severity. The severity of psoriasis and treatment response were evaluated using validated clinical and patient-reported outcome measures, specifically the PASI, IGA, and the DLQI. All scores were documented at baseline (T0) and after 2 months of topical intervention (T2). Two dermatologists independently assessed each patient and recorded PASI, DLQI, and IGA scores. The data were then compared, and a consensus-based objective score was documented for each patient. Outcome assessors were not blinded to the evaluation time point (baseline vs. follow-up), a limitation of the study.

### 2.9. Statistical Analysis

Statistical analysis was conducted using Jamovi software (version 2.7.2). Continuous variables were compared using the paired-samples Student’s *t*-test when the assumption of normality was met. In cases where the Shapiro–Wilk test indicated a deviation from normality, the nonparametric Wilcoxon signed-rank test was employed. When the Shapiro–Wilk normality test indicated a violation of the normality assumption, the Wilcoxon signed-rank test was employed. The results are presented as mean ± standard deviations, with effect sizes calculated using Cohen’s d and corresponding 95% confidence intervals (95% CI). Two-tailed *p*-values < 0.05 were considered statistically significant. Linear regression analysis was conducted to identify potential predictors of treatment responsiveness. Spearman’s rank correlation was performed as an exploratory analysis to assess the relationship between pre- and post-treatment PASI values and the inflammatory biomarkers.

## 3. Results

### 3.1. Pharmacological Study: Composition and Properties of the Lavender Extract

#### 3.1.1. Content of Organic Acids in the Lavender Extract

The bioactive constituents identified in the lavender extract are presented in Table 2.

Additional results from the analyses are provided in the Supplementary Materials (Figures S1–16).

#### 3.1.2. Organoleptic Evaluation

Organoleptic evaluation is an essential step in assessing the quality of a bioadhesive formulation, as it allows the examination of appearance, color, odor, homogeneity, and consistency of the final product. In the case of the bioadhesive formulation containing ethanolic lavender extract, this evaluation enabled the assessment of both physical and sensory stability, as well as the adequacy of the formulation and preparation process.

The organoleptic analysis confirmed that the bioadhesive formulation had a homogeneous appearance with no phase separation, a uniform olive-green color, a pleasant lavender-like odor without oxidative notes, and a soft, emollient consistency that was easily spreadable on the skin. These characteristics indicate good compatibility between the excipients (petrolatum, eucerin, and paraffin oil) and the ethanolic lavender extract, and support the physicochemical stability of the formulation.

#### 3.1.3. pH Determination

pH determination represents a fundamental step in evaluating the quality of the investigated bioadhesive formulation, as pH directly influences physicochemical stability, the activity of active compounds, and, most importantly, skin compatibility. For the formulated bioadhesive preparation, pH assessment is essential to confirm that the product is non-irritating upon topical application.

An appropriate pH contributes to formulation stability, prevents degradation of individual components, and ensures dermatological safety. pH measurements were performed at regular intervals (0, 7, 14, 21, 30, and 60 days) to monitor chemical stability and skin compatibility over time. Maintaining pH within the physiologic range (4.5–6.0) indicates the absence of component degradation processes and supports the conclusion that the formulation remains non-irritating and safe for topical use.

Longitudinal analysis of these parameters is essential for identifying potential stability-related changes, such as oxidation, phase separation, rancidity, or pH fluctuations, which may affect therapeutic efficacy and sensory acceptability. Consequently, the preservation of consistent organoleptic characteristics and a stable pH for 60 days demonstrates the stability, quality, and skin compatibility of the lavender extract-based topical formulation during storage. The main organoleptic characteristics of the topical formulation are summarized in Table 3.

#### 3.1.4. Rheological Characterization of the Bioadhesive Formulation

The rheological data showed a progressive decrease in viscosity with increasing shear rate from 1 to 100 s^−1^. This trend indicates non-Newtonian flow behavior, characteristic of semisolid systems, in which resistance to flow decreases with increasing shear stress.

The observed reduction in viscosity with increasing shear rate indicates shear-thinning (pseudoplastic) behavior, a common property of ointment formulations. From a functional perspective, this rheological profile is advantageous: at low shear rates (resting conditions), higher viscosity contributes to product stability and consistency, whereas at high shear rates (during application), reduced viscosity facilitates spreading and uniform distribution over the skin surface.

**Figure 5:**
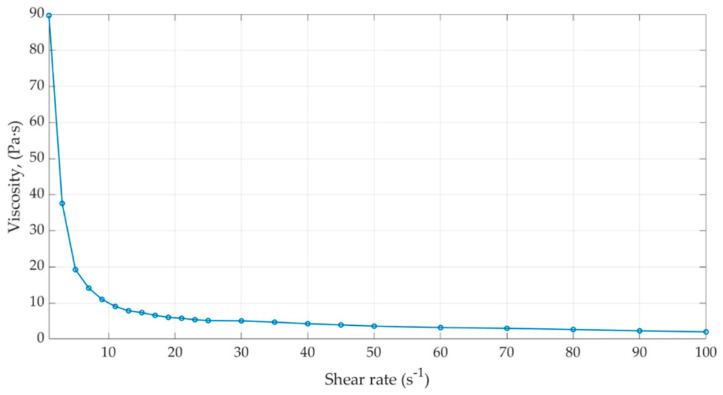
Viscosity characterization of the lavender extract-based formulation.

The rheological behavior can be attributed to the formulation composition. Petrolatum and Eucerin contribute to the semisolid structure and high viscosity at rest, while paraffin oil promotes fluidization under shear. The soft lavender extract may further influence the matrix microstructure, thereby contributing to the formulation’s overall rheological response (Figure 5).

#### 3.1.5. Colloidal Stability of the Bioadhesive Pharmaceutical Preparations

The complex composition of the bioadhesive formulation, as well as the preparation procedure, may affect colloidal stability and alter the formulation’s integrity. The proposed formulation exhibited high colloidal stability, with a stability index (SI) of 100%. The emulsifying agent played a key role in maintaining formulation stability. Eucerin, a lipophilic excipient with emulsifying properties, stabilizes the interfacial boundary between phases and supports the overall consistency of the formulation.

The uniformity of the contents was assessed by visual examination of a small amount of the preparation spread on a glass coverslip. No differences in color uniformity, phase separation, or non-uniform appearance were observed.

### 3.2. Clinical Study

#### 3.2.1. Study Population

##### The General Characteristics of the Patients

The patients in the study ranged from 28 to 71 years, with a mean age of 52.5 ± 13.26 years. Male patients (83.33%) were predominant (*p* = 0.008) and were slightly older than females (53.33 ± 14.41 vs. 48.33 ± 3.22 years, *p* = 0.567). The duration of CKD ranged from 2 to 30 years, with a mean of 7.73 ± 7.038 years. The main underlying renal condition was chronic glomerulonephritis (CGN), followed by tubulointerstitial nephritis (TIN). The duration of CKD monitoring ranged from 2 to 15 years. Among associated comorbidities, arterial hypertension was present in 17 of 18 patients, and obesity in 8 patients. Regarding the histopathological subtype of CGN, this was identified in five patients: three with IgA nephropathy, one with a heterozygous Alport syndrome variant combined with Wilson’s disease, and one with membranoproliferative glomerulonephritis. At the time of the study, none of the patients with glomerulonephritis required immunosuppressive treatment. They were receiving antiproteinuric treatment with angiotensin-converting enzyme inhibitors (ACEi) or angiotensin II receptor blockers (ARB). The study population had a mean age of 52.5 ± 13.26 years (Table 4).

#### 3.2.2. Renal-Related Laboratory Parameters Throughout the Study

Three patients were undergoing maintenance hemodialysis at baseline. Among the remaining participants, eGFR values ranged from 25 to 115 mL/min/1.73 m^2^. Serum uric acid was available for 14 patients (range: 4–10.1 mg/dL). Urinary albumin creatinine ratio (uACR) was assessed longitudinally in 14 patients, with values ranging from 26 to 979 mg/g. Throughout the study, the majority of patients remained at the same CKD stage, while one patient improved from stage G1A2 to G1A1 (Table 5).

#### 3.2.3. The Inflammatory Biomarkers Measured Throughout the Study Are Presented in Table S1 (Supplemental Files)

##### The Efficacy of the Triterpenoids Isolated from *Lavandula angustifolia* Extract

**Figure 6:**
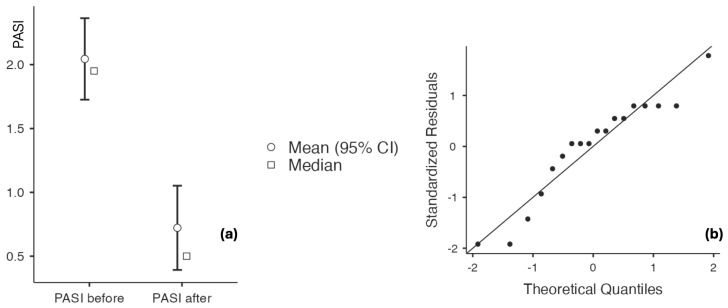
(**a**) PASI at baseline and after 60 days of topical triterpenoid extract application; (**b**) Q-Q plot: assessment of residuals.

**Figure 7:**
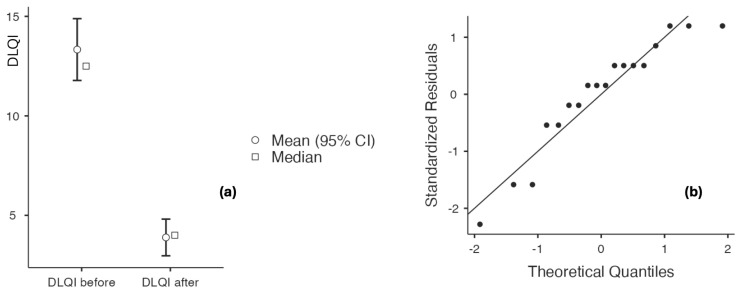
(**a**) DLQI at baseline and after 60 days of topical triterpenoid extract application; (**b**) Q-Q plot: assessment of residuals.

**Figure 8:**
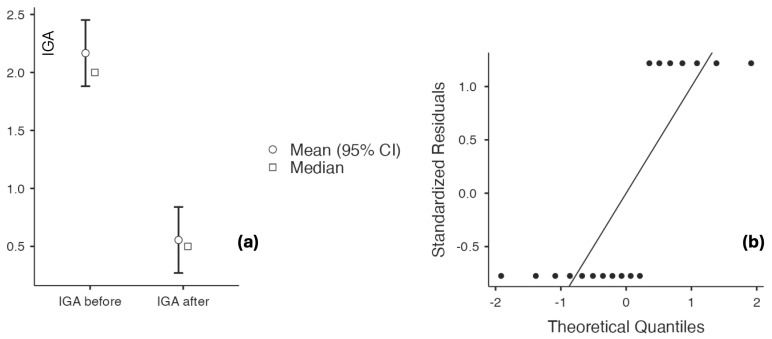
(**a**) IGA at baseline and after 60 days of topical triterpenoid extract application. (**b**) Q-Q plot: assessment of residuals.

**Figure 9:**
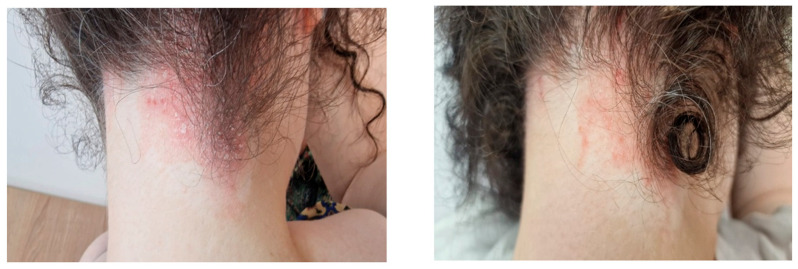
The clinical comparative results obtained after applying lavender extract preparations to patients.

The study population primarily had mild-to-moderate chronic plaque psoriasis, as determined by clinical severity assessment. The baseline scores for PASI, IGA, and DLQI consistently indicated low to moderate disease activity across the cohort, with no patients meeting criteria for severe psoriasis or necessitating systemic antipsoriatic treatment at the time of inclusion. The findings are shown in Table 6 and Figure 6, Figure 7, Figure 8 and Figure 9, indicating that psoriasis severity ameliorated following two months of topical treatment. The mean PASI decreased from 2.044 ± 0.690 at baseline to 0.722 ± 0.714 at follow-up (*p* < 0.001), and the mean IGA improved from 2.167 ± 0.618 to 0.556 ± 0.616 (*p* < 0.001). Simultaneously, quality of life was markedly enhanced, as evidenced by a reduction in DLQI from 13.333 ± 3.361 to 3.889 ± 1.997 (*p* < 0.001).

##### The Renal Safety of the Triterpenoids Isolated from *Lavandula angustifolia* Extract

**Figure 10:**
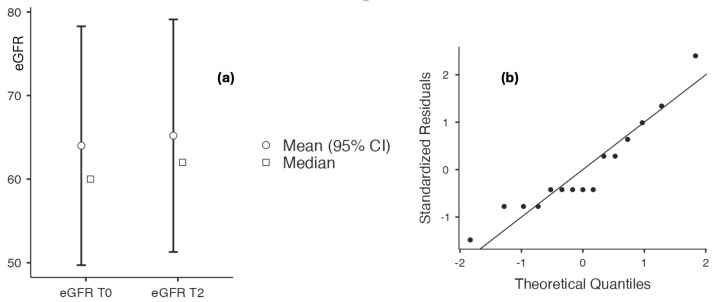
(**a**) eGFR at baseline and after 60 days of topical triterpenoid extract application. (**b**) Q-Q plot: assessment of residuals.

**Figure 11:**
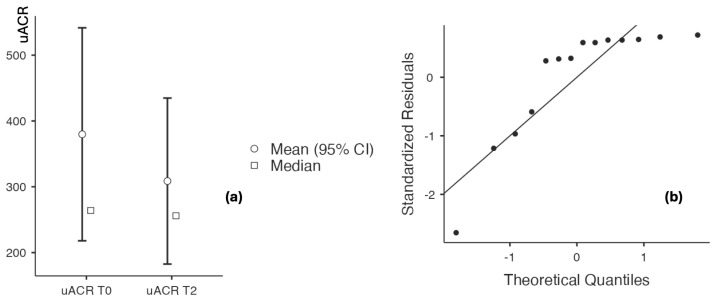
(**a**) uACR at baseline and after 60 days of topical triterpenoid extract application. (**b**) Q-Q plot: assessment of residuals.

The comparative evaluation of creatinine, eGFR, and uACR values was performed using Student’s *t*-test when data followed a normal distribution, and the Wilcoxon test when normality was violated, as assessed by the Shapiro–Wilk test. Serum creatinine levels remained similar throughout the study period (1.5 ± 0.668 vs. 1.467 ± 0.646, *p* = 0.327). Changes in eGFR were evaluated using the Wilcoxon test and were not statistically significant (65.2 ± 27.483 vs. 64 ± 28.229, *p* = 0.123). No significant differences were observed in serum uric acid levels (6.192 ± 1.915 vs. 6.715 ± 1.815, *p* = 0.974) or hemoglobin levels (14.272 ± 1.810 vs. 14.444 ± 1.861, *p* = 0.326). The only parameter that showed a statistically significant difference was uACR, with lower values at T2 compared to T0 (308.714 ± 240.782 vs. 379.78 ± 308.81, *p* = 0.001) (Table 7 and Figure 10 and Figure 11).

##### Assessment of Inflammatory Status Before and After Application of the Topical Triterpenoid Cream

**Figure 12:**
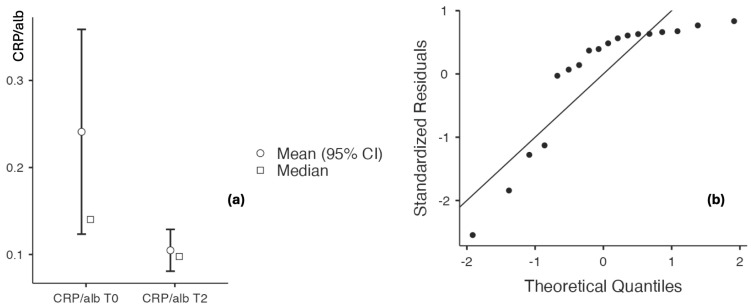
(**a**) hs-CRP/albumin at baseline and after 60 days of topical triterpenoid extract application. (**b**) Q-Q plot: assessment of residuals.

**Figure 13:**
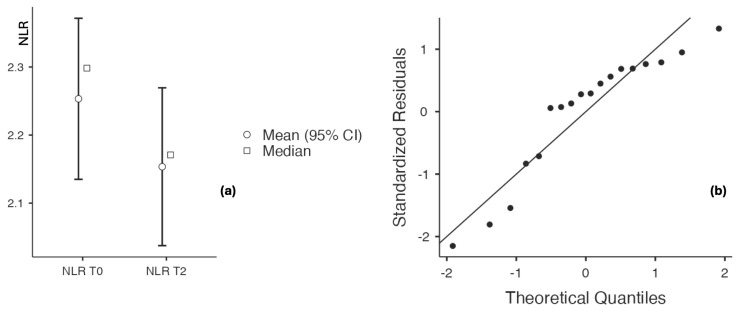
(**a**) NLR at baseline and after 60 days of topical triterpenoid extract application. (**b**) Q-Q plot: assessment of residuals.

Evaluation of inflammatory biomarkers before and after application of the triterpenoid-containing cream demonstrated a decreasing trend. Hs-CRP levels decreased from 9.7 ± 10.045 to 4.33 ± 2.127 (*p* = 0.009), while the CRP/albumin ratio declined from 0.241 ± 0.225 to 0.105 ± 0.052 (*p* = 0.011), with no significant change observed in serum albumin levels (4.106 ± 0.218 vs. 4.167 ± 0.233, *p* = 0.156). The neutrophil-to-lymphocyte ratio (NLR) also decreased significantly, from 2.253 ± 0.256 to 2.154 ± 2.171 (*p* < 0.001), with a large effect size of 0.988 (Table 8 and Figure 12 and Figure 13).

##### Assessment of Predictors of Positive Response to Topical Triterpenoid Therapy in the Treatment of Psoriasis

In an attempt to identify predictors of positive response to topical triterpenoid therapy, baseline inflammatory biomarkers were initially considered. However, the proposed models, analyzed individually for hs-CRP, hs-CRP/albumin ratio, and NLR, did not confirm the predictive value of these biomarkers for responsiveness to the topical treatment. In the case of NLR, baseline value was not significantly associated with delta PASI (ß = −0.249, 95%CI [−0.336–0.680], *p* = 0.534, adjusted R^2^ 0.025). The diagnostic indicated that the model was stable: Cook’s distance was low (0.061), and the variation inflation factor was 1.0, confirming the absence of multicollinearity. Residuals appeared approximately normally distributed, and no model assumption violations relevant to the study design were identified. For hs-CRP, the regression coefficient was ß =−0.007 (95%CI [−0.692–0.353], *p* = 0.504), and for the hsCRP/albumin ratio, ß =−0.239 (95%CI [−0.674–0.374], *p* = 0.552) (Table 9).

**Figure 14:**
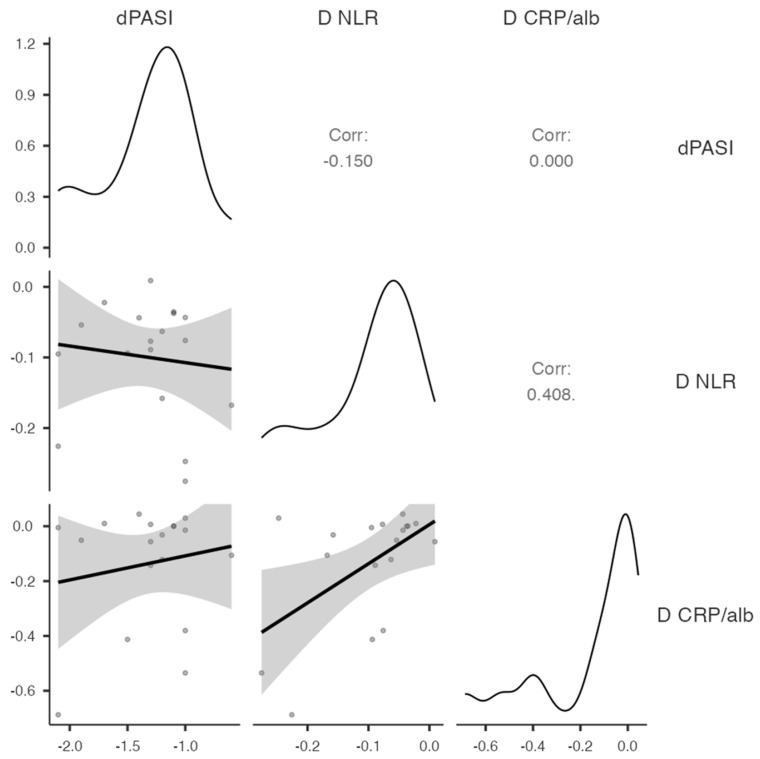
Exploratory Spearman correlation analyses for ΔPASI, ΔNLR and Δhs-CRP/albumin ratio.

Given the lack of significant baseline predictors, an exploratory analysis was performed to examine the changes(Δ) in PASI, CRP/albumin ratio, and NLR. Exploratory Spearman correlation analyses revealed no significant associations between changes in PASI score and changes in inflammatory markers. Specifically, ΔPASI was not significantly correlated with ΔNLR (ρ = 0.15, *p* = 0.277) or ΔCRP/albumin ratio (ρ = 0.00, *p* = 0.500), and changes in CRP/albumin ratio and NLR were also not significantly correlated (ρ = 0.41, *p* = 0.954). These results suggest that, within this small sample, modifications in systemic inflammatory markers were not associated with changes in treatment response (Figure 14).

## 4. Discussion

Psoriasis is a chronic immune-mediated inflammatory condition with a high prevalence and a steady upward trend [45]. Treatment options for psoriasis include systemic, phototherapy, and topical therapies. Local treatment is the first choice for mild-to-moderate psoriasis because it is applied directly to the affected area, bypassing the first hepatic passage, with controlled and flexible dosing, but most importantly for patients, rapid efficacy. However, their effectiveness is often limited by poor penetration of active substances into the skin [46]. Psoriatic plaque is not only associated with inflammation of the epidermis, but also extends to the dermis as a result of uncontrolled proliferation of keratinocytes via TNF-α, IL-17, and IFN-γ. In T-cell-mediated plaque psoriasis, the TNF-α-IL-23-Th17 inflammatory pathway is characteristic [47]. Numerous studies indicate that reactive oxygen species (ROS) and nitric oxide synthases (NOSs) are involved in the pathogenesis of psoriasis. Oxidative stress triggers protein kinases (MAPKs) and nuclear factor kappa-light-chain enhancer of activated B cells (NF-kB), leading to Th cell activation, inflammatory cytokine release, and cell proliferation [48,49,50].

Microalbuminuria is a relevant biomarker of endothelial dysfunction at the glomerular level and a marker of systemic inflammation. It has been demonstrated that chronic inflammatory diseases are associated with the onset or progression of microalbuminuria. As a chronic, relapsing, inflammatory skin disorder, psoriasis shares common pathogenic pathways with microalbuminuria, including the involvement of pro-inflammatory cytokines, oxidative stress, and disturbances in nitric oxide metabolism and its derivatives [51]. Although psoriasis’s association with cardiovascular disease and diabetes mellitus is well recognized, its renal involvement remains insufficiently clarified. Studies demonstrate that microalbuminuria correlates with psoriasis duration, with PASI, and with the presence of psoriatic arthritis [52].

Of the total patients who received topical antipsoriatic therapy, 18 individuals were identified in the records of Pelican Hospital Oradea, across various departments, including Diabetology, Internal Medicine, Cardiology, and Pneumology. The mean age at the time of evaluation was 52.5 ± 13.26 years, with the youngest patient being 28 years old, who had a 6-year history of CKD and a diagnosis of congenital solitary kidney. In our study, the majority of patients were male (83.33%).

Recent studies have demonstrated that sex and age influence the distribution, treatment response, and potential complications of psoriasis, highlighting the importance of these parameters in guiding therapeutic strategies [53].

Chronic glomerulonephritis (CGN) was the predominant underlying renal pathology in these patients, with IgA nephropathy identified as the main histopathological entity. Numerous studies have reported that IgA nephropathy is associated with psoriasis [54,55]. However, detailed genetic studies indicate that among inflammatory skin diseases, only atopic dermatitis is associated with IgA nephropathy [56]. Obesity, by promoting a chronic pro-inflammatory milieu, is a key factor influencing both the onset and progression of psoriasis. In our cohort, 44.44% of patients were obese, representing a clinically relevant prognostic indicator for disease severity in psoriasis and for the progression of chronic kidney disease. The functional component of CKD, as reflected by the eGFR calculated according to KDIGO 2021 recommendations using the CKD-EPI creatinine equation, did not exhibit statistically significant changes over the study period (maximum follow-up 7 months: 60 days pre-treatment, treatment period, and 60 days post-treatment).

For most patients, eGFR declined slightly and nonsignificantly, so participants largely remained within their pre-treatment CKD stages. This observation may reflect a reduction in systemic inflammatory activity, as indicated by the hs-CRP/albumin ratio and NLR, which showed a trend toward post-treatment decreases.

Microalbuminuria, a marker of glomerular injury, improved over the course of the study. However, most patients were hypertensive and receiving treatment with ACE inhibitors or ARBs, making it impossible to establish a definitive link between the observed trend in microalbuminuria, the progression of kidney disease, and the topical use of triterpenoids.

With the exception of one patient, all participants were on renin–angiotensin–aldosterone system (RAAS) blockade therapy, either with ACE inhibitors or ARBs.

These findings suggest that topical antipsoriatic therapy with lavender extract, in addition to its cutaneous efficacy, is associated with preserved renal function and stabilization of glomerular injury markers and can be administered safely in patients with CKD and psoriasis without detectable renal adverse effects.

The therapeutic efficacy of pharmaceutical preparations is closely related to the treated condition, the physicochemical properties of the active compounds, and, importantly, the characteristics of excipients and formulation design, which influence the release of the active ingredient across the dosage form. In topical formulations, these factors also determine skin permeability and local bioavailability [57,58]. An alternative to conventional topical therapies containing synthetic drugs are plant-based topical formulations, which exhibit anti-inflammatory and antioxidant effects and are associated with a reduced incidence of adverse reactions.

Oleanolic acid is a bioactive compound widely distributed in numerous medicinal plants and exhibits a broad spectrum of pharmacological activities, including antidiabetic, hepato- and neuroprotective, antiviral, anti-inflammatory, and antibacterial effects, as demonstrated by multiple research groups [59,60,61,62]. The anti-inflammatory activity of oleanolic acid (OA) has been extensively evaluated in experimental studies using laboratory animals.

According to Dong et al., OA administration led to a significant downregulation of cyclooxygenase-2 (COX-2), inducible nitric oxide synthase (iNOS), and major pro-inflammatory cytokines, including interleukin-1β (IL-1β), IL-6, and tumor necrosis factor-α (TNF-α), in *Salmonella typhimurium*-infected mice, concomitant with preservation of intestinal barrier integrity and improvement of diarrheal symptoms [63]. At the cardiac level, Martin et al. demonstrated that OA administration in mice reduced cytokine-induced calcium and collagen deposition associated with myocarditis. Furthermore, OA administration was associated with a significant increase in the production of the anti-inflammatory cytokines IL-10 and IL-35, accompanied by a marked decrease in pro-inflammatory cytokine levels [64]. Furthermore, Wang et al. demonstrated the antioxidant activity of OA in rat models by activating nuclear factor erythroid 2-related factor 2 (Nrf2), mitogen-activated protein (MAP) kinases, and modulation of cellular redox responses [65].

Ursolic acid is a naturally occurring pentacyclic triterpenoid with well-documented antioxidant, anti-inflammatory, and anticancer properties. The anti-inflammatory activity of ursolic acid (UA) is primarily attributed to its ability to suppress NF-κB activation. UA has also been shown to exert anti-inflammatory effects by markedly inhibiting the secretion of cytokines such as IL-2, IL-4, IL-6, and interferon gamma (IFN-γ) when immune cells are treated with 5 μM UA. Similar effects were observed in CD4+ T lymphocytes. Importantly, UA exhibited no cytotoxic effects on lymphocytes, as assessed by propidium iodide (PI) staining [66,67].

Pomolic acid exhibits predominantly cytotoxic effects, which have been associated with increased caspase-3 and caspase-9 activity, as well as enhanced reactive oxygen species (ROS) production [68]. Rosmarinic acid is a naturally occurring phenolic compound with potent antioxidant, anti-inflammatory, anticancer, and antimicrobial properties. Its antioxidant and anti-inflammatory activities are largely mediated through inhibition of NF-κB signaling. Administration of rosmarinic acid at 40 mg/kg in rat models of chronic induced lesions resulted in a significant reduction in inflammatory markers, including prostaglandin E2 (PGE2), IL-1β, and COX-2 [69]. Additionally, a concentration of 5 μM significantly inhibited IL-4 and IFN-γ production in activated CD4+ T cells [70].

Also, ursolic acid and oleanolic acid act as agonists of the nuclear PPARα receptors [71]. Activation of these receptors inhibits the maturation and function of dendritic cells via the SIRT1/NF-κB pathway, cells that play a fundamental role in the pathogenesis of psoriasis [72].

Although the beneficial effects of these bioactive compounds have been demonstrated in numerous studies, their use in topical therapy remains limited due to their hydrophobic nature. The therapeutic efficacy of such active compounds largely depends on the formulation in which they are incorporated, as formulation design can enhance or limit skin penetration and consequently modulate biological activity. A properly designed formulation base therefore plays a crucial role in determining the overall effectiveness of pharmaceutical preparations [73].

Bio-adhesive formulations increase the contact time between the incorporated active ingredient and the skin surface, thereby enhancing local bioavailability through sustained release from the cross-linked matrix structure. Additionally, these formulations promote skin hydration by forming a thin hydrophilic film on the skin surface and reducing transepidermal water loss, both of which are essential for skin regeneration. The rheological properties of the formulation further facilitate application and support penetration of the active compounds into deeper skin layers. Cosmetic attributes—including color, odor, non-greasy skin feel, and overall appearance—should not be overlooked, as these characteristics significantly influence patient acceptance and treatment adherence [74].

In the analyzed cohort, psoriasis severity was predominantly classified as mild-to-moderate based on standardized clinical assessment. This distribution reflects real-world clinical practice, particularly in patients with chronic kidney disease, for whom systemic therapeutic options are often limited due to comorbidities and safety considerations. In this context, topical therapy remains a cornerstone of psoriasis management, and objective quantification of clinical response and quality-of-life impact is essential.

The concordance observed between PASI, IGA, and DLQI scores supports a relatively homogeneous clinical profile among the included patients, characterized by limited skin involvement but persistent symptoms with a measurable impact on quality of life. This finding underscores the clinical relevance of psoriasis even in mild-to-moderate forms, especially in vulnerable populations with associated chronic conditions.

In line with these observations, the results indicate a clinically meaningful improvement after two months of topical treatment. The IGA score improved from a median value of 2.0 (IQR 2.0–2.75) to 0.5 (IQR 0.0–1.0), while the PASI score decreased from a median of 1.95 (IQR 1.63–2.55) to 0.50 (IQR 0.00–1.38). Furthermore, the DLQI score declined from 12.5 (IQR 11.0–15.75) to 4.0 (IQR 2.25–5.0), indicating a substantial reduction in the perceived disease burden among patients.

The study has several limitations, including a small sample size, heterogeneous demographic characteristics, a short duration of topical treatment application, and limited generalizability of the results to the broader population. There was no control group in the study’s single-arm design, which limits the ability to draw causal conclusions and leaves open the possibility of regression to the mean or placebo effects. Moreover, there were no intermediate time points; assessments were conducted only at baseline and day 60. Furthermore, the precise dose per unit of skin surface area (g/cm^2^) was not quantitatively recorded, despite the use of a practical fingertip-unit-based dosing instruction for topical administration. This could affect inter-patient reproducibility. Chronic kidney disease stages varied widely, ranging from G1 to G5, and uACR values spanned a broad spectrum. Due to the single-arm design, small sample size, and limited quantitative dosing standardization, these results should be regarded as preliminary and hypothesis-generating. Although the study included a relatively small number of patients, the results may complement the existing research base and support future larger-scale studies aimed at developing a topical preparation for use as a complementary therapy in patients with psoriasis.

The study also presents several novel aspects. The isolation of bioactive compounds from lavender was performed using a method designed to prevent degradation, with controlled sourcing of the vegetal material. The bioactive principles were incorporated into a novel topical formulation, which was systematically evaluated for organoleptic characteristics, irritability, stability, rheological properties, and therapeutic efficacy. The stability of the topical preparation over 60 days was monitored at repeated intervals (7, 14, 21, 30, and 60 days). The absence of changes in appearance, color, and pH, along with the lack of phase separation, indicates good stability and the absence of possible interactions between the components of the mixture. This formulation was prepared in small quantities at regular intervals, according to each patient’s needs and the size of the affected area. The efficacy of the topical preparation is supported by the improved appearance of the psoriasis-affected area and favorable changes in the monitored systemic inflammatory markers. Although the efficacy of lavender extract-containing preparations has been investigated by multiple research groups, the bioactive compounds described in those studies were derived primarily from the volatile oil and differ from those evaluated in the present work [75,76]. In contrast, the four bioactive compounds incorporated into our topical preparation were identified and quantified after removal of the volatile oil.

The patient cohort included in the clinical study represents a niche population characterized by complex pathology and an additional pro-inflammatory burden [77,78,79]. From a theoretical perspective, the demonstration of favorable outcomes in this population suggests that these results may be extrapolated and potentially exhibit even greater efficacy in the general psoriasis population. Furthermore, using combined inflammatory biomarkers expressed as composite indices enhances the clinical relevance of these observations in these patients by integrating the nutritional component into the chronic inflammatory profile.

This study is the first to demonstrate the safety of topical lavender extract administration in patients with CKD. Given the observed outcomes, our findings warrant further investigation in larger, population-based cohorts and longer-term follow-up to evaluate relapse prevention and confirm both the local and systemic effects of the applied product.

## 5. Conclusions

Psoriasis is a disease with a complex pathogenesis and an increasing global incidence. It affects patients of all ages and has a substantial impact on quality of life due to its visible manifestations, associated pain, pruritus, and overall discomfort. The development of topical formulations containing natural active compounds showing potential clinical benefit and favorable tolerability offers new therapeutic opportunities for managing psoriasis in patients with chronic kidney disease (CKD). However, the promising effects of their application on inflammatory status and microalbuminuria, a marker of endothelial dysfunction, require confirmation in larger patient populations. The lavender extract-based topical formulation developed in this study exhibited appropriate organoleptic characteristics, favorable viscosity and skin adhesion, good physicochemical stability, and a pH value of 5.5, without inducing skin irritation. These findings support the potential of this bio-adhesive formulation as a safe and well-tolerated topical option for symptom management in patients with mild to moderate psoriasis, particularly in populations with limited systemic treatment options. Larger randomized trials with standardized topical dosing and intermediate assessments are needed to confirm efficacy and better characterize the dynamics of treatment response over time.

## Figures and Tables

**Table 1:** Composition of the bioadhesive topical formulation containing lavender extract.

Formulation Composition	Amount (%)	Function in the Formulation
Ethanolic extract of *Lavandula angustifolia* (L.)	3.50 g	Pharmacologically active ingredient with antiseptic, anti-inflammatory, soothing, and wound-healing properties
Paraffin oil	10.00 g	Emollient; dispersing agent for the bioactive compound
Eucerin	30.00 g	Emulsifying ointment base
White petrolatum	56.50 g	Solid lipid base

**Table 2:** The lavender extract yield and the content of organic acids in the extract.

Plant Dry Weight, g	Extract Mass, g	Content of Organic Acid, % Extract			
		OA	UA	PA	RA
100	12.4	3.6	8.0	3.6	1.4

**Table 3:** Organoleptic parameters and pH values of the bioadhesive formulation over time.

Evaluated Parameter	Initial	7 Days	14 Days	21 Days	30 Days	60 Days
Centrifugation	No phase separation	No phase separation	No phase separation	No phase separation	No phase separation	No phase separation
Appearance	Homogeneous	Homogeneous	Homogeneous	Homogeneous	Homogeneous	Homogeneous
Color	Olive green	Olive green	Olive green	Olive green	Olive green	Olive green
Odor	Characteristic	Characteristic	Characteristic	Characteristic	Characteristic	Characteristic
pH	5.50	5.50	5.50	5.50	5.50	5.50

**Table 4:** The main characteristics of the patients.

	Age 52.5 ± 13.26	Sex/M 83.3%	History of CKD 7.73 ± 7.04	Kidney Disease	Observations
1	32	M	30	TIN	Reflux nephropathy; HT
2	47	F	2	CGN	Alport syndrome; Wilson disease; HT
3	46	M	5	CGN	IgA nephropathy; HT
4	64	M	15	ADPKD	Diabetes mellitus; HT; Obesity
5	52	M	8	CGN	MPGN; HT
6	28	M	6	TIN	Unique congenital left kidney; HT Obesity
7	46	F	3	TIN	Vitiligo; Hypothyroidism; Microscopic hematuria
8	68	M	3	DN	Diabetes mellitus; Cirrhosis; HT Obesity
9	46	M	2	CGN	IgA nephropathy; HT; Obesity
10	68	M	4	VN	COPD; HT; Obesity
11	44	M	15	CGN	IgA nephropathy; HT
12	62	M	3	VN	Renal cyst; HT; Obesity
13	54	M	3	CGN	Diabetes mellitus; HT
14	71	M	11	VN	Obesity; HT
15	68	M	6	TIN	Right pneumectomy; HT
16	52	F	3	TIN	HT; Multiple liver adenoma
17	34	M	3	CGN	Polycythemia; HT
18	63	M	10	CGN	IgA nephropathy; HT; Obesity

Legend: M—male; F—Female; HT—arterial hypertension; COPD—chronic obstructive pulmonary disease; MPGN—membranoproliferative glomerulonephritis; TIN—tubulointerstitial nephropathy; ADPKD—autosomal dominant polycystic kidney disease; VN—vascular nephropathy.

**Table 5:** Renal-related laboratory parameters for patients’ study.

No	Creatinine (mg/dL)		eGFR (mL/min/1.73 m^2^)		Uric Acid (mg/d)		uACR (mg/g)		CKD Stage	
	**T0**	**T2**	**T0**	**T2**	**T0**	**T2**	**T0**	**T2**	**T0**	**T2**
1	1.98	2	45	45	7.5	10.1	110	98	G3aA2	G3aA2
2	0.82	0.84	89	86	4	4.1	979	795	G2A3	G2A3
3	1.45	1.36	60	65	N/A	N/A	682	521	G2A3	G2A3
4	2.74	2.76	25	25	4.6	4.6	207	196	G4A2	G4A2
5	HD	HD			3.5	6.5	N/A	N/A	G5	G5
6	2.7	2.7	32	32	8.5	8.8	621	580	G3bA3	G3bA3
7	0.8	0.81	92	91	4.5	4.7	42	26	G1A2	G1A1
8	HD	HD			N/A	N/A	N/A	N/A	G5	G5
9	2.25	1.88	36	44	7.6	4.7	821	503	G3bA3	G3bA3
10	1.44	1.43	53	53	5.9	N/A	N/A	N/A	G3a	G3a
11	0.86	0.87	109	109	4.7	4.9	529	487	G1A3	G1A3
12	1.8	1.74	38	42	7.7	5.7	318	306	G3bA3	G3bA3
13	HD				N/A	6.7	N/A	N/A	G5	G5
14	1.58	1.54	46	48	6.3	7.6	78	33	G3a	G3a
15	1.24	1.26	63	62	N/A	5.5	154	147	G2	G2
16	0.85	0.83	82	85	6.8	5.1	54	38	G2	G2
17	0.91	0.89	113	115	5.7	N/A	210	206	G1	G1
18	1.08	1.09	77	76	8.4	7.5	512	386	G2	G2

Legend: HD—hemodialysis; N/A—not available; eGFR—estimated glomerular filtration rate; CKD—chronic kidney disease; GxAx—CKD staging according KDIGO; T0 = baseline; T2 = after 60 days.

**Table 6:** Changes in psoriasis severity and quality-of-life outcomes after 2 months of topical treatment.

Parameter	T0	T2	*p*	ES	95%CI	Shapiro–Wilk
PASI	2.044 ± 0.690	0.722 ± 0.714	<0.001 ^†^	3.263 ^#^	2.075–4.432	*p* = 0.120
DLQI	13.333 ± 3.361	3.889 ± 1.997	<0.001 ^†^	3.286 ^#^	2.090–4.466	*p* = 0.069
IGA	2.167 ± 0.618	0.556 ± 0.616	<0.001 *	1 ^§^		*p* < 0.001

T0 = baseline; T2 = after 60 days; ES—effect size; *—Wilcoxon rank; ^†^—paired *t*-test; ^§^—rank biserial; ^#^—Cohen’s d effect size.

**Table 7:** Changes in renal disease laboratory analysis after 2 months of topical triterpenoid treatment.

Parameter	T0	T2	*p*	ES	95%CI	Shapiro–Wilk
Creatinine mg/dL	1.5 ± 0.668	1.467 ± 0.646	0.327 *	0.305 ^§^		*p* < 0.001
eGFR mL/min/1.73 m^2^	64 ± 28.229	65.2 ± 27.483	0.123 ^†^	−0.424 ^#^	−0.946–0.131	*p* = 0.119
uACR mg/g	379.78 ± 308.81	308.714 ± 240.782	0.001 ^†^	1 ^§^		*p* < 0.001
Uric acid mg/dL	6.715 ± 1.815	6.192 ± 1.915	0.974 ^†^	−0.010 ^#^	−0.575–0.557	*p* = 0.708
Hemoglobin g/L	14.444 ± 1.861	14.272 ± 1.810	0.326	0.319 ^§^		*p* < 0.001

T0 = baseline; T2 = after 60 days; ES—effect size; *—Wilcoxon rank; ^†^—paired *t*-test; ^§^—rank biserial; ^#^—Cohen’s d effect size.

**Table 8:** Comparative inflammatory biomarkers evaluation before after 2 months of topical treatment.

Parameter	T0	T2	*p*	ES	Shapiro–Wilk
Hs-CRP mg/dL	9.7 ± 10.045	4.33 ± 2.127	0.009 *	0.725 ^§^	*p* < 0.001
Albumin g/dL	4.106 ± 0.218	4.167 ± 0.233	0.156	−0.451 ^§^	*p* = 0.007
CRP/albumin mg/g	0.241 ± 0.225	0.105 ± 0.052	0.011	0.712 ^§^	*p* < 0.001
NLR	2.253 ± 0.256	2.154 ± 2.171	<0.001	0.988 ^§^	*p* = 0.027

T0 = baseline; T2 = after 60 days; ES—effect size; *—Wilcoxon rank; ^§^—rank biserial.

**Table 9:** Linear logistic regression, independently for hs-CRP, hs CRP/albumin ratio and NLR.

Parameter	ß	95%CI	*p*	R^2^	Cooks Distance	VIF	RMSE
Hs CRP	−0.007	−0.692–0.353	0.502	0.029	0.137 ± 0.384	1	0.388
CRP/albumin	−0.239	−0.674–0.374	0.552	0.023	0.129 ± 0.341	1	0.389
NLR	−0.249	−0.366–0.680	0.534	0.025	0.061 ± 0.091	1	0.389

## Data Availability

The original contributions presented in this study are included in the article/Supplementary Material. Further inquiries can be directed to the corresponding author.

## Supplementary Materials

The following supporting information can be downloaded at: https://www.mdpi.com/article/10.3390/biomedicines14030552/s1, Figure S1. ^1^H NMR spectrum of oleanolic acid; Figure S2. ^13^C NMR spectrum of oleanolic acid; Figure S3. DEPT NMR spectrum of oleanolic acid; Figure S4. HSQC NMR spectrum of oleanolic acid; Figure S5. ^1^H NMR spectrum of ursolic acid; Figure S6. ^13^C NMR spectrum of ursolic acid; Figure S7. DEPT NMR spectrum of ursolic acid; Figure S8. HSQC NMR spectrum of ursolic acid; Figure S9. ^1^H NMR spectrum of pomolic acid; Figure S10. ^13^C NMR spectrum of pomolic acid; Figure S11. DEPT NMR spectrum of pomolic acid; Figure S12. HSQC NMR spectrum of pomolic acid; Figure S13. ^1^H NMR spectrum of rosmarinic acid; Figure S14. ^13^C NMR spectrum of rosmarinic acid; Figure S15. DEPT NMR spectrum of rosmarinic acid; Figure S16. HSQC NMR spectrum of rosmarinic acid containing internal standard methyl 4-nitrobenzoate; Table S1: Biomarkers of inflammation at baseline and after 60 days.
