# Supplementary material for: Prospective Pilot Study of Lavender Terpenoid Topical Formulation in Psoriasis Patients with Chronic Kidney Disease: Clinical and Inflammatory Outcomes
Source: Biomedicines. 2026 Feb 27;14(3):552. doi: 10.3390/biomedicines14030552 (PMC13024350; doi:10.3390/biomedicines14030552)
Supplement: Supplementary file 1 [file biomedicines-14-00552-s001.zip › biomedicines-4170974-supplementary.pdf]

Table S 1. Biomarkers of inflammation at baseline and after 60 days

No	Hs-CRP (mg/dl)		albumin		neutrophils		lymphocytes		Hs-CRP/albumin		NLR	
	T0	T2	T0	T2	T0	T2	T0	T2	T0	T2	T0	T2
1	7	2.9	4	4.2	6100	5400	3100	3000	0.175	0.069	1.968	1.800
2	5.6	5.3	3.9	4.1	5600	5300	2700	2610	0.144	0.129	2.074	2.031
3	1	1	4.2	4.2	5800	5710	2400	2400	0.024	0.024	2.417	2.379
4	4.9	4.8	4.1	3.8	6170	5700	2850	2730	0.120	0.126	2.165	2.088
5	27	7.6	3.7	3.9	6730	5870	2900	2870	0.730	0.195	2.321	2.045
6	2	2.5	3.8	4	6800	6750	3200	3210	0.053	0.063	2.125	2.103
7	4.2	3.9	4.1	4	5300	4950	2700	2650	0.102	0.098	1.963	1.868
8	36	8.2	4.1	4.3	6700	5400	3110	2800	0.878	0.191	2.154	1.929
9	3.7	1.5	4.3	4.3	7400	7250	3200	3210	0.086	0.035	2.313	2.259
10	10	8	4.2	4.4	7150	7050	2860	2810	0.238	0.182	2.500	2.509
11	7.9	2.8	4.3	4.5	7320	7030	3180	3140	0.184	0.062	2.302	2.239
12	1.3	3.1	4.4	4.2	6400	6100	2789	2710	0.030	0.074	2.295	2.251
13	22	5.9	3.9	3.9	5920	5730	2510	2530	0.564	0.151	2.359	2.265
14	2.9	3	4.2	4.3	7510	6980	3190	3010	0.069	0.070	2.354	2.319
15	3	4.4	4.4	4.5	7450	7210	2510	2650	0.068	0.098	2.968	2.721
16	5.2	4	3.8	3.8	7530	7290	3100	3210	0.137	0.105	2.429	2.271
17	9.4	3.2	4.4	4.5	5800	5720	3100	3210	0.214	0.071	1.871	1.782
18	21.5	5.9	4.1	4.1	6740	6520	3400	3420	0.524	0.144	1.982	1.906

Supplementary material

NMR spectra of organic acids determined in lavender extracts

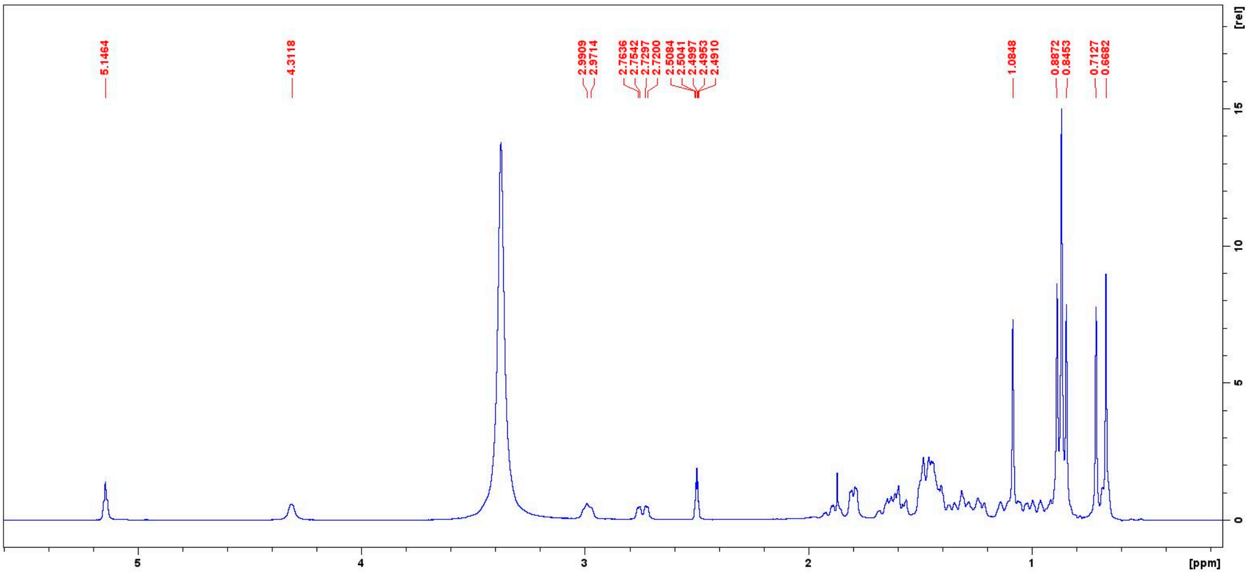

Figure S1. ^{1}H NMR spectrum of oleanolic acid

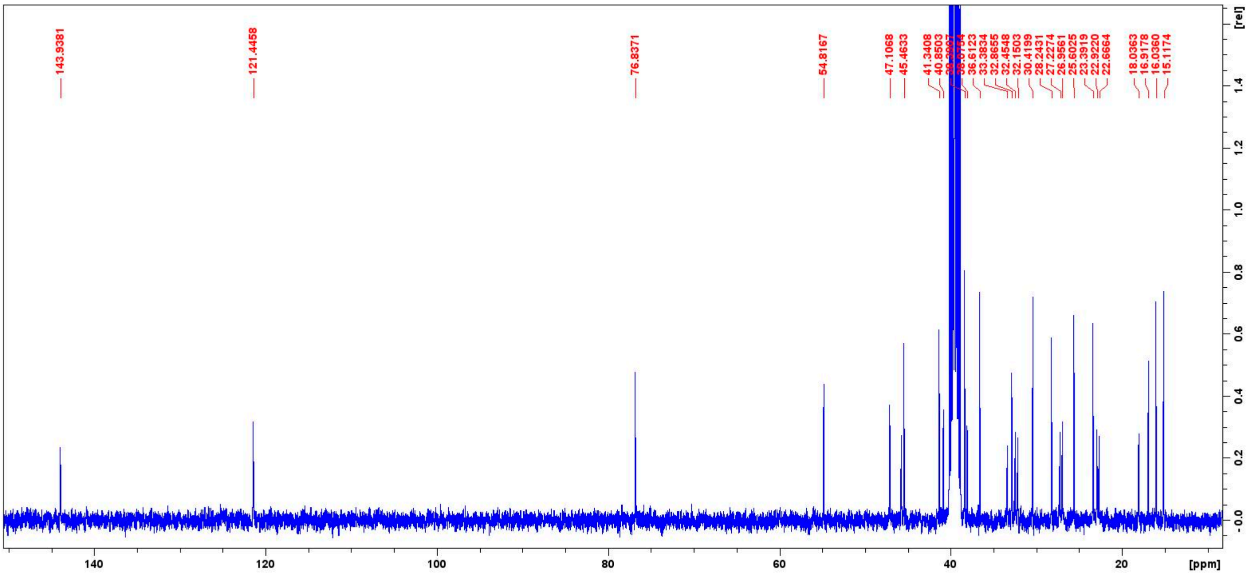

Figure S2. ^{13}C NMR spectrum of oleanolic acid

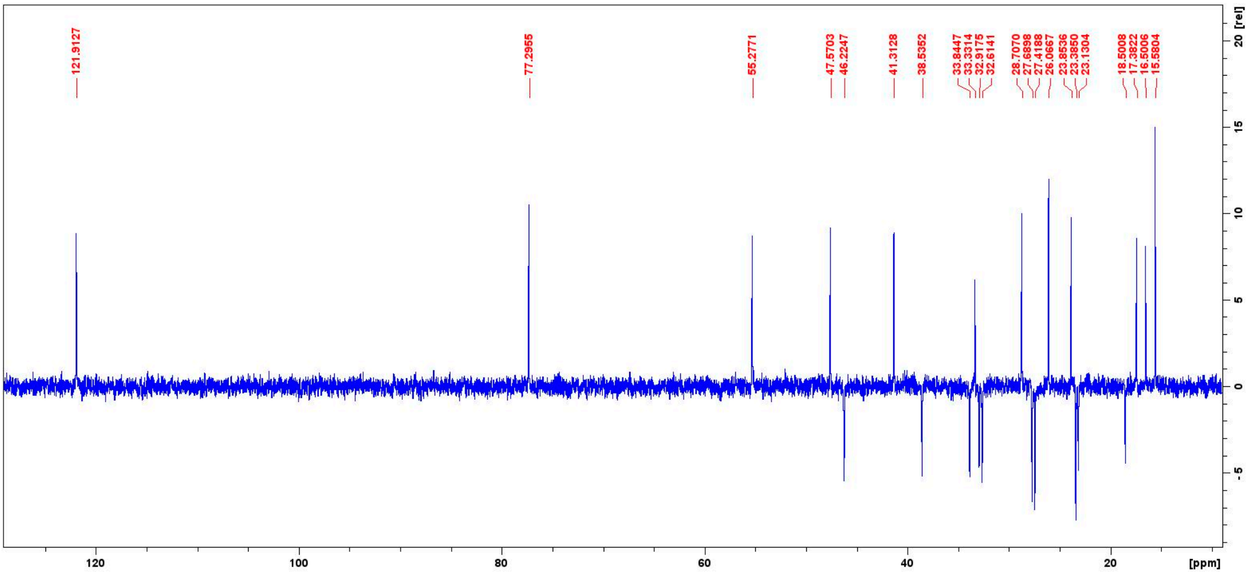

Figure S3. DEPT NMR spectrum of oleanolic acid

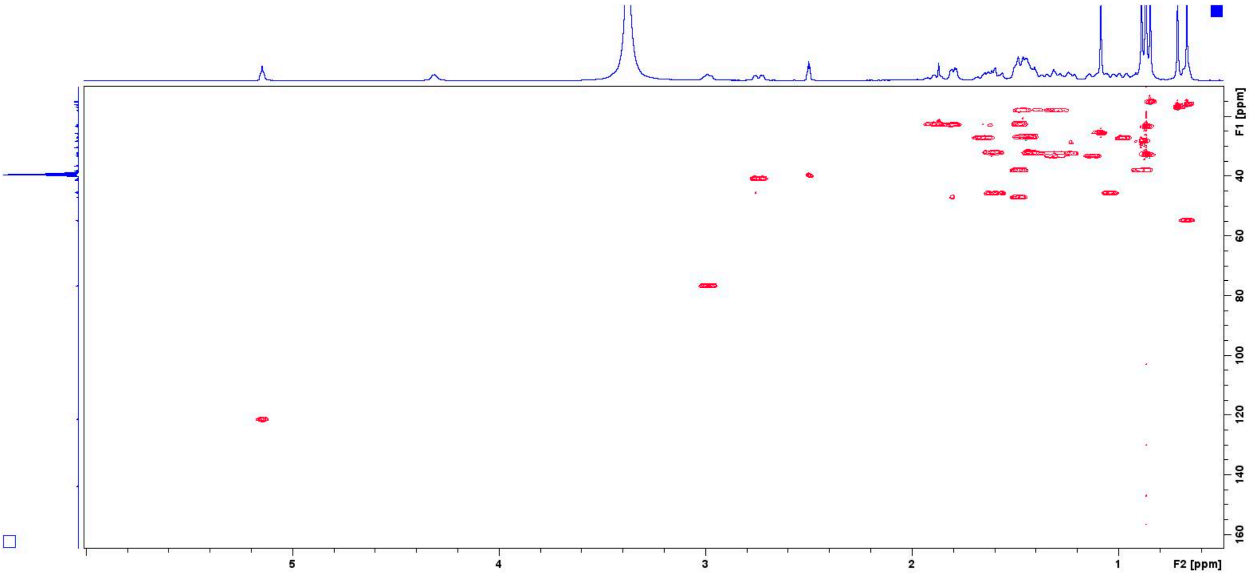

Figure S4. HSQC NMR spectrum of oleanolic acid

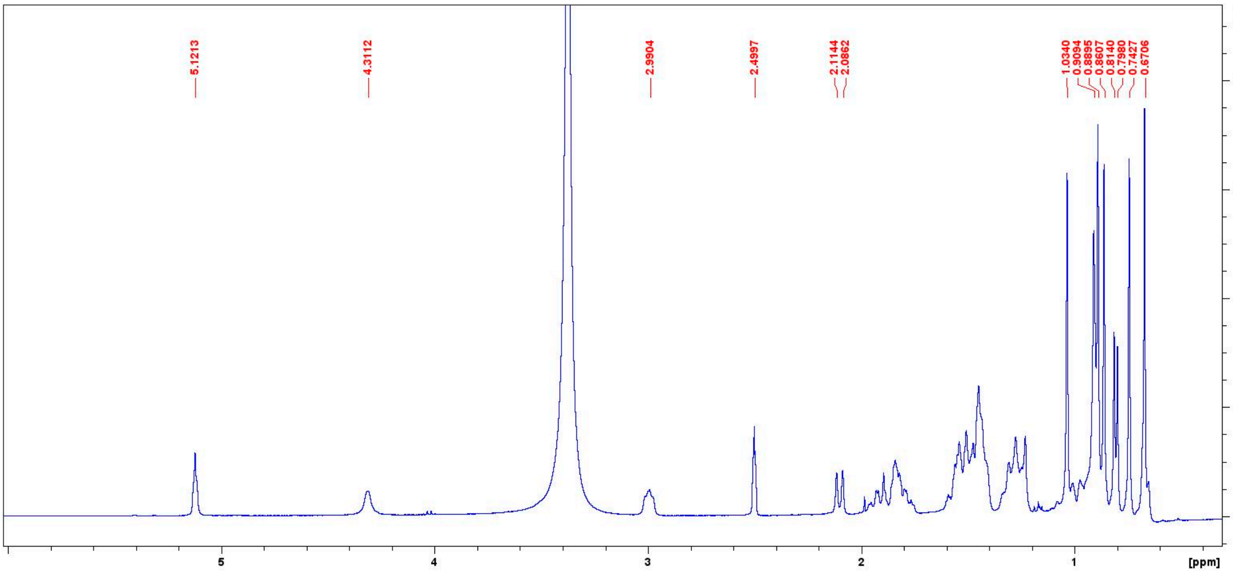

Figure S5. ^{1}H NMR spectrum of ursolic acid

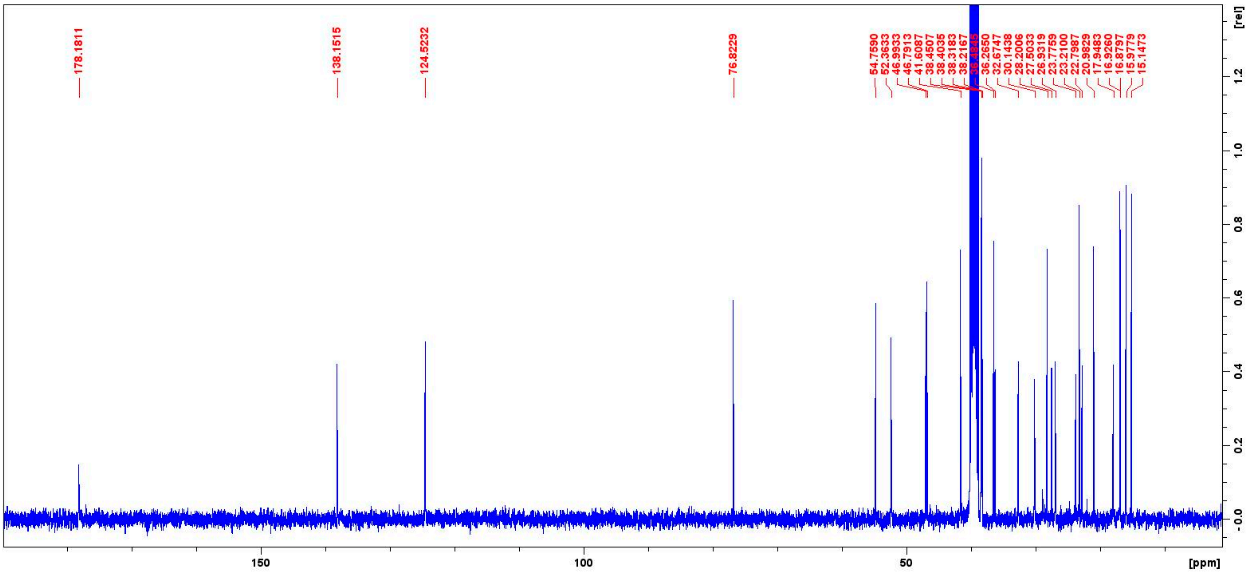

Figure S6. ^{13}C NMR spectrum of ursolic acid

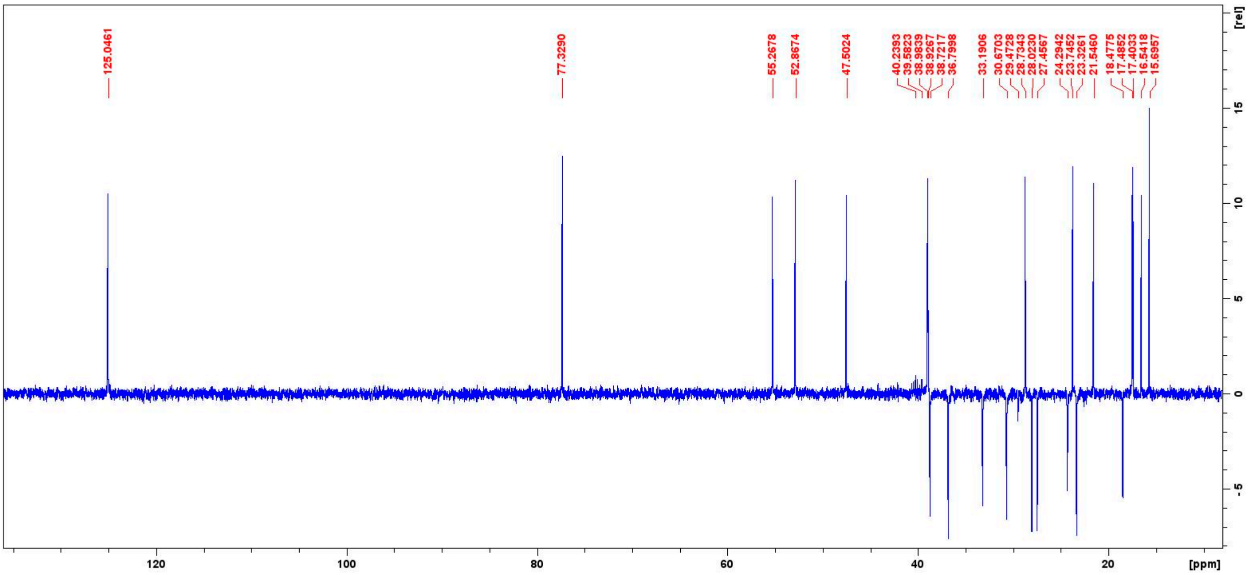

Figure S7. DEPT NMR spectrum of ursolic acid

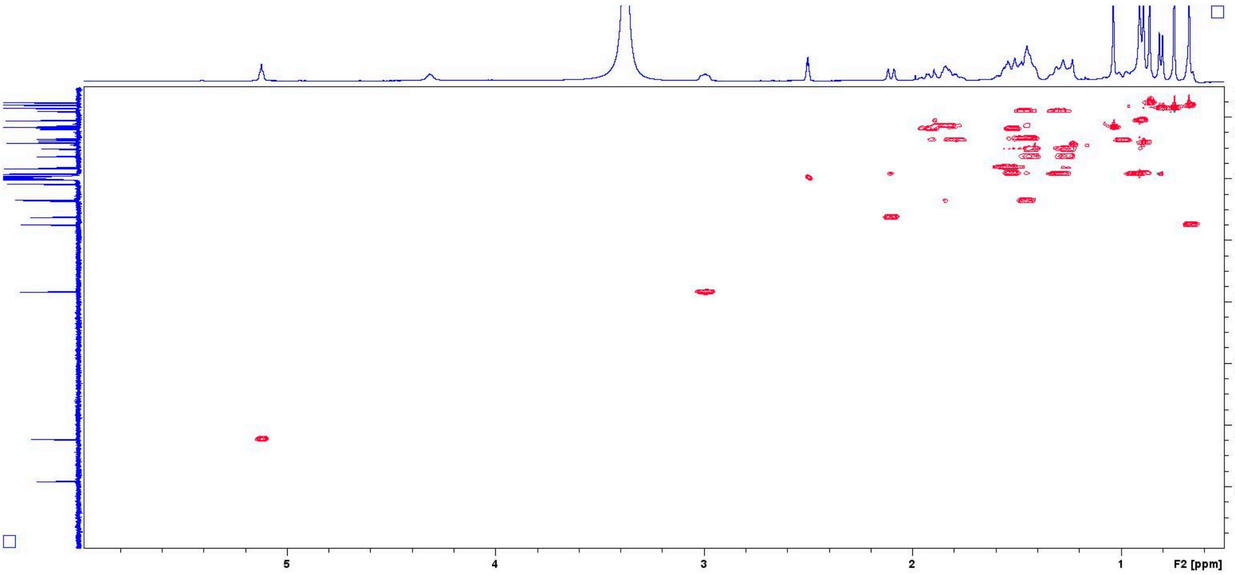

Figure S8. HSQC NMR spectrum of ursolic acid

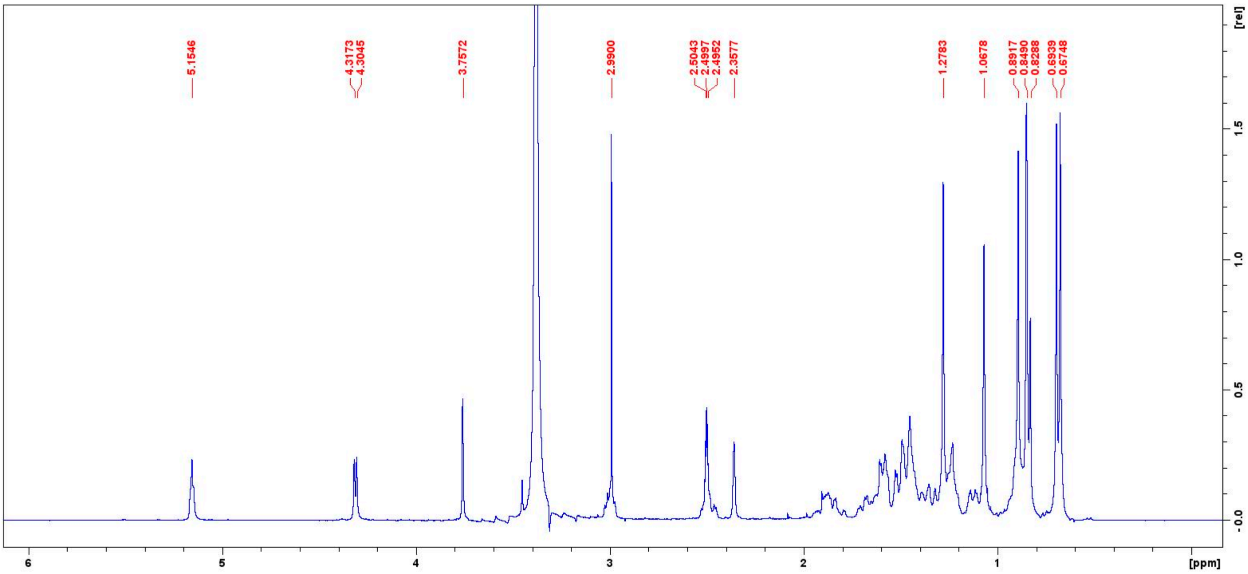

Figure S9. ^{1}H NMR spectrum of pomolic acid

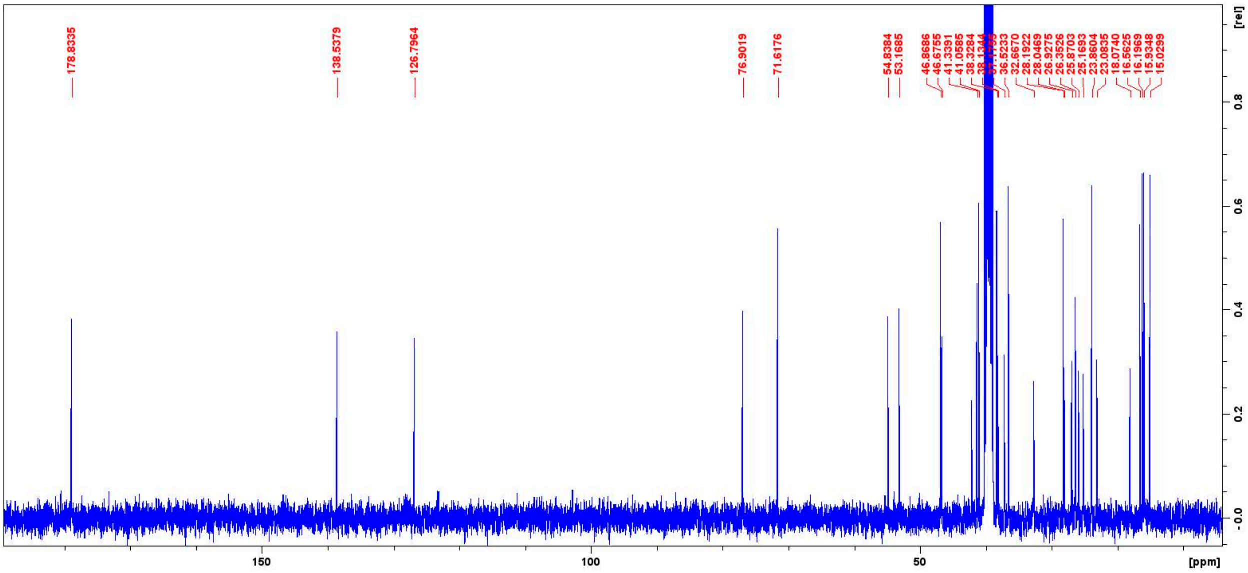

Figure S10. ^{13}C NMR spectrum of pomolic acid

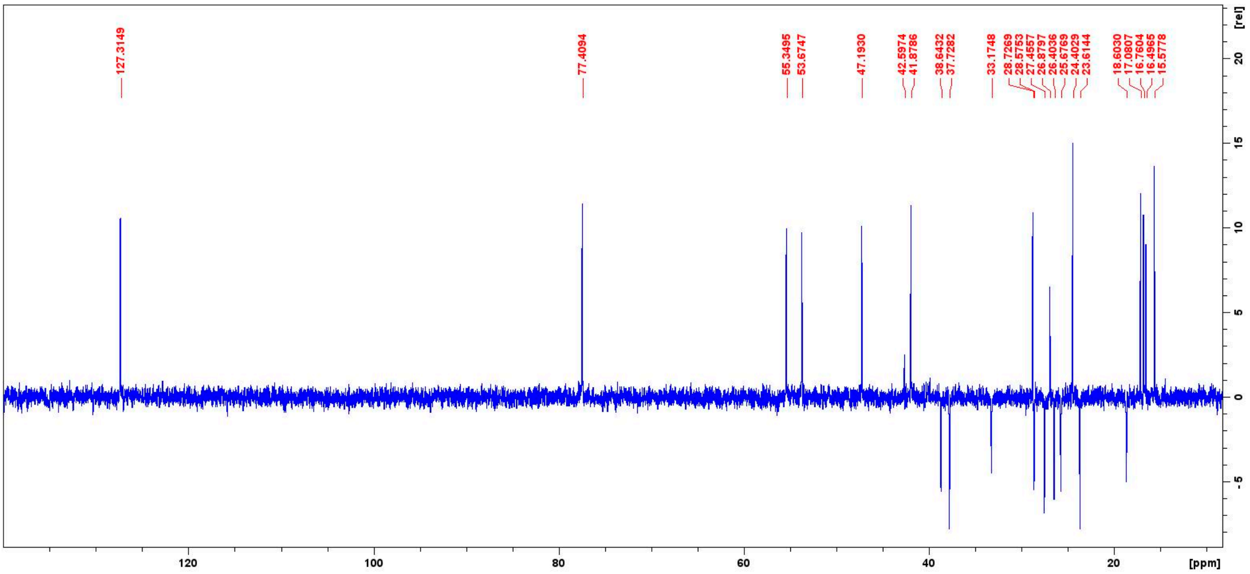

Figure S11. DEPT NMR spectrum of pomolic acid

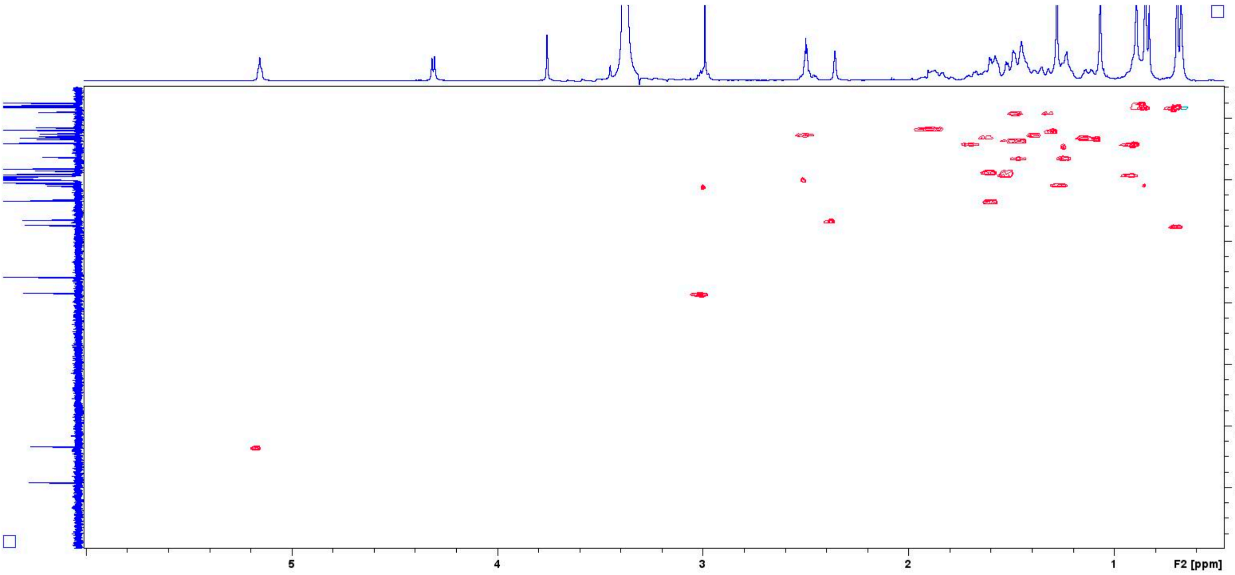

Figure S12. HSQC NMR spectrum of pomolic acid

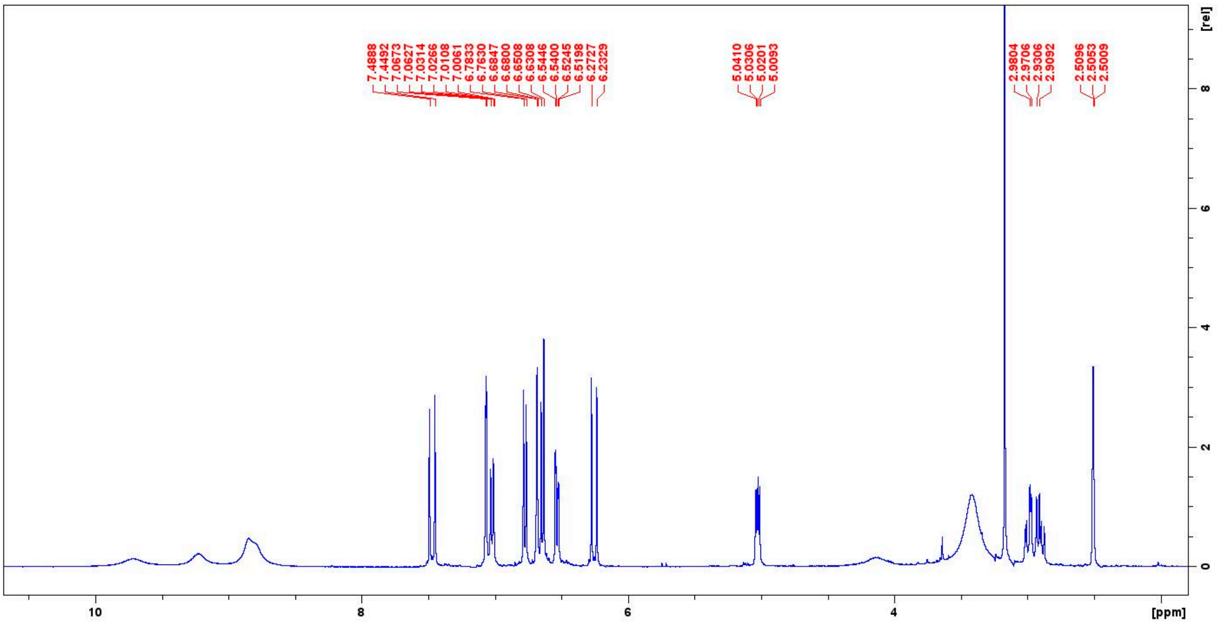

Figure S13. ^{1}H NMR spectrum of rosmarinic acid

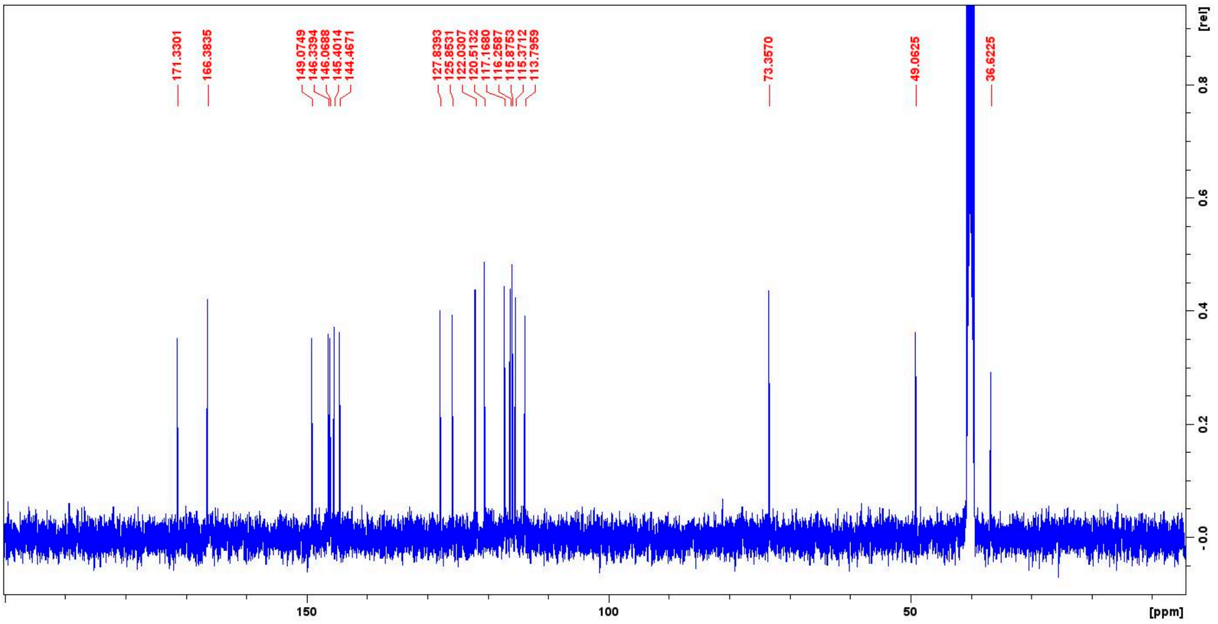

Figure S14. ^{13}C NMR spectrum of rosmarinic acid

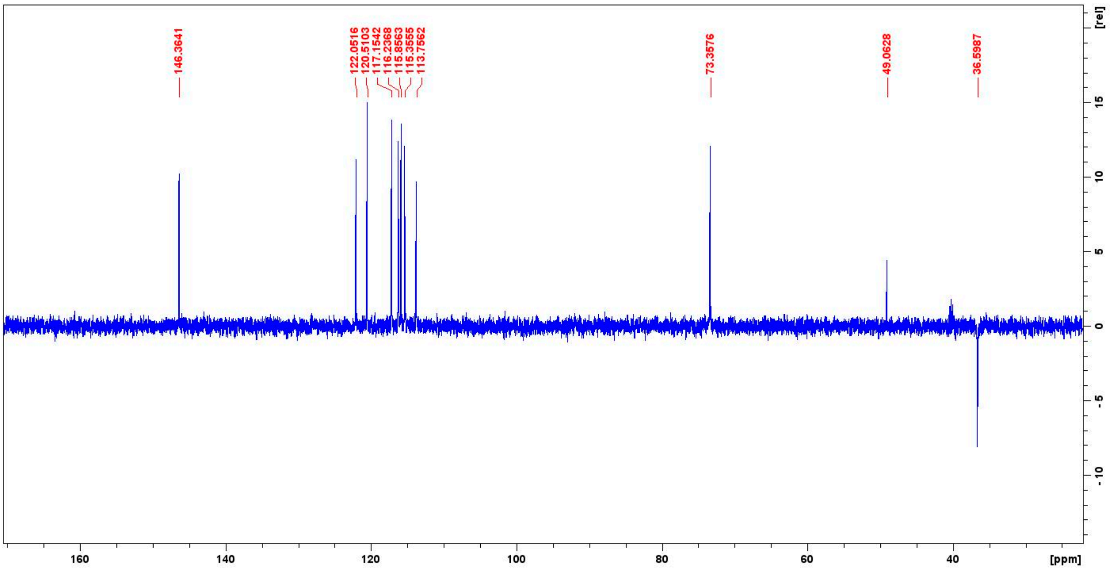

Figure S15. DEPT NMR spectrum of rosmarinic acid

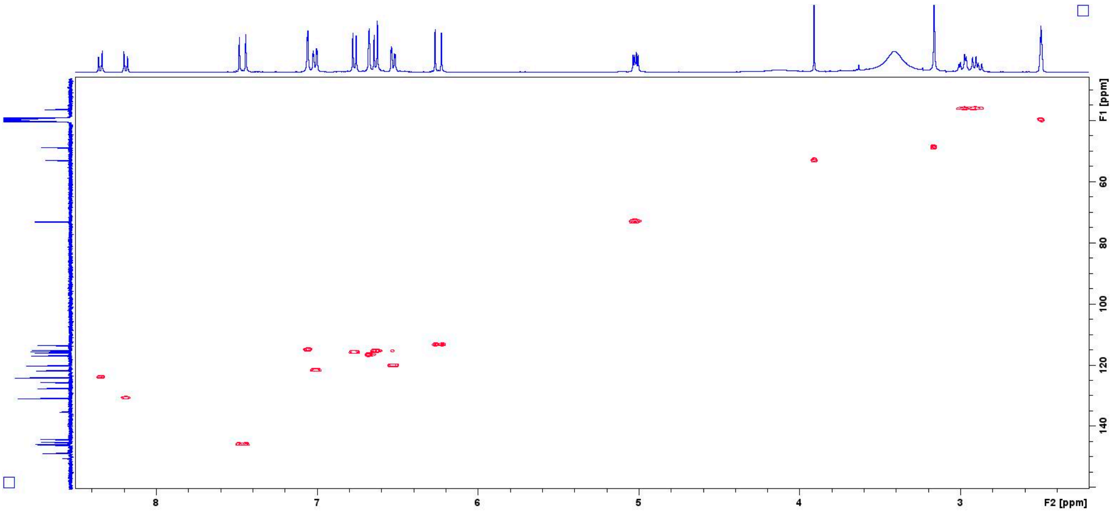

Figure S16. HSQC NMR spectrum of rosmarinic acid containing internal standard – methyl 4-nitrobenoate
